# Structural prerequisites for G-protein activation by the neurotensin receptor

**DOI:** 10.1038/ncomms8895

**Published:** 2015-07-24

**Authors:** Brian E. Krumm, Jim F. White, Priyanka Shah, Reinhard Grisshammer

**Affiliations:** 1 Membrane Protein Structure Function Unit, National Institute of Neurological Disorders and Stroke, National Institutes of Health, Department of Health and Human Services, 5625 Fishers Lane, Rockville, Maryland 20852, USA

## Abstract

We previously determined the structure of neurotensin receptor NTSR1 in an active-like conformation with six thermostabilizing mutations bound to the peptide agonist neurotensin. This receptor was unable to activate G proteins, indicating that the mutations restricted NTSR1 to relate agonist binding to G-protein activation. Here we analyse the effect of three of those mutations (E166A^3.49^, L310A^6.37^, F358A^7.42^) and present two structures of NTSR1 able to catalyse nucleotide exchange at Gα. The presence of F358^7.42^ causes the conserved W321^6.48^ to adopt a side chain orientation parallel to the lipid bilayer sealing the collapsed Na^+^ ion pocket and linking the agonist with residues in the lower receptor part implicated in GPCR activation. In the intracellular receptor half, the bulkier L310^6.37^ side chain dictates the position of R167^3.50^ of the highly conserved D/ERY motif. These residues, together with the presence of E166^3.49^ provide determinants for G-protein activation by NTSR1.

## Abstract (web summary)

The structural basis of how G-protein coupled receptors respond to unique stimuli remains poorly understood. Here, Krumm *et al.* present new structures of the neurotensin receptor and reveal insights into how ligand binding is linked to structural rearrangements associated with receptor activation.

## Graphical abstract

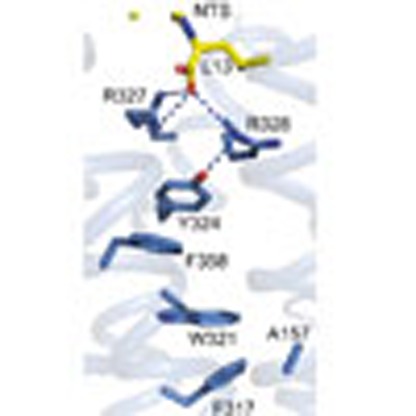

GPCRs are highly versatile signalling molecules that modulate second messenger responses in the cell. Binding of an extracellular agonist leads to conformational changes in the receptor, triggering activation of associated signalling partners on the intracellular side of the membrane. GPCRs are no longer thought to be two-state switches (inactive or active, although rhodopsin may come close to this definition) but are able to sample many conformational states depending on the bound ligand, associated signalling partner and membrane environment^1^. Recent advances in the structural biology of GPCRs have resulted in high-resolution snapshots of inactive^2^, active-like^3^ and active receptor conformations^4^,^5^,^6^,^7^.

Our research focuses on understanding the structural and functional requirements for the activation of the neurotensin receptor 1 (NTSR1, ref. 8). Its agonist ligand neurotensin (NTS) is a 13-amino acid peptide that functions as a neurotransmitter and a hormone in the nervous system and in peripheral tissues^9^. NTS has a wide range of biological activities with important aspects in antinociception, cancer cell growth and the pathogenesis of schizophrenia^10^,^11^. Most of the known agonist effects of NTS are mediated through NTSR1 (refs 8, 11).

Previously, we determined the structure of NTSR1 bound to NTS_8–13_ (Arg-Arg-Pro-Tyr-Ile-Leu) in an active-like conformation with six thermostabilizing mutations providing insight into the binding mode of a peptide agonist. This receptor was unable to catalyse nucleotide exchange at the Gα subunit, indicating that some of the stabilizing mutations may have restricted the ability of NTSR1 to relate agonist binding to the activation of G protein. Here we analyse the effect of three of those six mutations (E166A^3.49^, L310A^6.37^, F358A^7.42^) on G-protein activation and present structures of active-like NTSR1 that are able to activate G protein. Unique to these structures is the presence of a phenylalanine at position 7.42 causing the conserved W321^6.48^ to adopt a side chain rotamer conformation parallel to the lipid bilayer, which has not been seen in any GPCR structures to date. The W321^6.48^ residue seals the top of the collapsed Na^+^ ion-binding pocket and along with the F358^7.42^ residue links the agonist peptide, bound closer to the extracellular surface, with residues in the lower part of NTSR1 that are implicated in conformational changes for GPCR activation. In the intracellular receptor half, the bulkier L310^6.37^ side chain dictates the position of R167^3.50^ of the highly conserved D/ERY motif. This, together with the presence of the neighbouring E166^3.49^ provides determinants for G-protein activation.

## Results

### NTSR1 constructs used in this study

Here, we describe structural, biochemical and pharmacological data of several NTSR1 mutants (see Supplementary Tables 1 and 2) with either wild-type intracellular loop 3 (ICL3), or with most of ICL3 replaced by T4 lysozyme (T4L). In the Methods section, we distinguish between NTSR1 constructs containing the wild-type ICL3 sequence or T4L. In the main text, we use only one name for a particular construct for ease of reading. For example, NTSR1-ELF refers interchangeably to NTSR1-ELF-T4L and NTSR1-ELF, the latter containing the wild-type ICL3, not T4L. The identity of the respective construct is evident from the context of writing. Protein crystals were obtained with receptors where most of ICL3 was replaced by T4L. Pharmacological and biochemical experiments were conducted with receptors containing the wild-type ICL3 sequence, but also included T4L variants for comparison. In Figures and Tables relating to biochemical and pharmacological data, the identity of constructs is unambiguously specified.

### Active-like NTSR1 mutants which activate Gq protein

**Figure 1:**
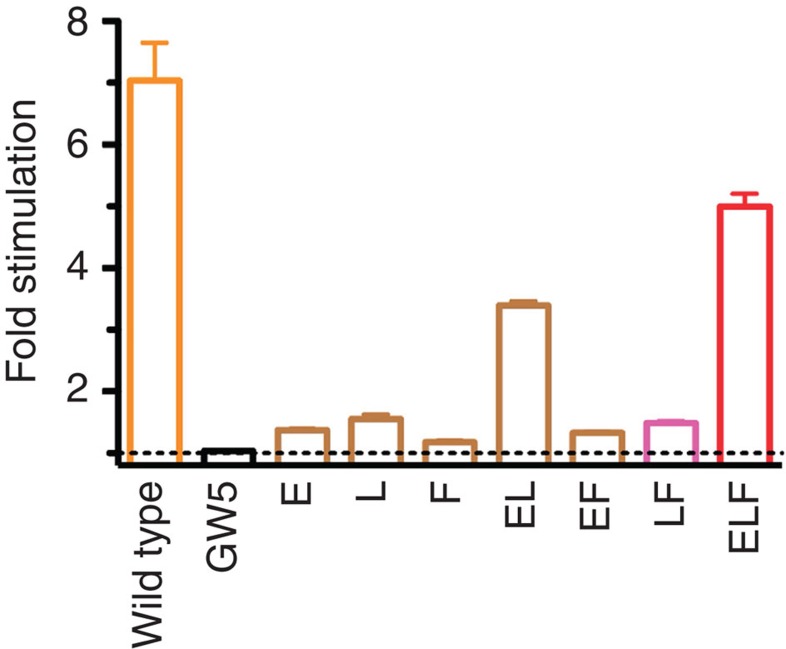
Mutational analysis of NTSR1 for activation of G protein. Agonist-stimulated activation of Gq: GDP/[^35^S]GTPγS exchange assays contained purified Gq protein, [^35^S]GTPγS, insect cell membranes with NTSR1 and saturating concentrations of NTS (20 μM). Fold stimulation of the exchange of GDP for [^35^S]GTPγS at Gq in the presence of NTS is compared with the nucleotide exchange in the absence of ligand (number of independent experiments: wild-type NTSR1 *n*=7; NTSR1-GW5 *n*=1; NTSR1-E *n*=4; NTSR1-L *n*=5; NTSR1-F *n*=4; NTSR1-EL *n*=5; NTSR1-EF *n*=4; NTSR1-LF *n*=7; NTSR1-ELF *n*=6). A value of 1 (dotted line) indicates the absence of receptor-catalysed nucleotide exchange. All G-protein activation experiments were conducted with NTSR1 constructs (containing the wild-type ICL3, not T4L) in urea-washed P2 insect cell membranes. The identity of the NTSR1 constructs is given in Supplementary Table 1. Error bars correspond to s.e.m.

We previously reported the crystal structure of NTSR1-GW5 (ref. 3) containing six stabilizing mutations (A86L^1.54^, E166A^3.49^, G215A^ECL2^, L310A^6.37^, F358A^7.42^ and V360A^7.44^; ref. 12; superscripts are the Ballesteros–Weinstein numbers^13^). NTSR1-GW5 displayed features of an active-like receptor such as an outward-tilted transmembrane helix (TM) 6 at the cytoplasmic surface and key conserved residues in positions characteristic for active and/or active-like but not for inactive GPCRs. NTSR1-GW5 did not catalyse nucleotide exchange at Gαq in response to NTS in G-protein-coupling assays (Fig. 1)^3^, suggesting that some of the stabilizing mutations have limited the ability of NTSR1-GW5 to activate G protein. On the basif of their location, we assumed that the mutations E166A^3.49^, L310A^6.37^ and F358A^7.42^ affected the NTSR1 activation state, whereas the involvement of the mutations A86L^1.54^, G215A^ECL2^ and V360A^7.44^ was not obvious^3^. We therefore reverted the three mutations E166A^3.49^, L310A^6.37^ and F358A^7.42^ to wild-type residues, alone or in combination (Supplementary Table 1), to analyse their role in G-protein activation. In contrast to NTSR1-GW5, the triple revertant NTSR1-ELF (with E166, L310, F358) was able to stimulate nucleotide exchange at Gαq to almost wild-type level (Fig. 1, Supplementary Fig. 1, Supplementary Table 2). The double revertant NTSR1-EL (with E166 and L310) showed reduced activity in nucleotide exchange assays, but highlights the importance of E166^3.49^, of the highly conserved D/ERY motif, along with the neighbouring L310^6.37^ for G-protein activation (Fig. 1).

### Pharmacological characterization of NTSR1-LF and NTSR1-ELF

Ligand-binding experiments (Supplementary Table 2) showed that the apparent affinity of NTSR1-LF and NTSR1-ELF for the agonist NTS was comparable to that of the wild-type receptor and NTSR1-GW5. The agonist-binding experiments using wild-type NTSR1 were conducted at equilibrium. In contrast, binding of [^3^H]NTS to the NTSR1 mutants did not reach equilibrium within the incubation time because of the slow agonist off-rates. The apparent IC_50_ values for the antagonist SR48692 (ref. 14) were 3–5-fold higher than the corresponding wild-type value, but more than 20-fold lower than the value for NTSR1-GW5. The shift in IC_50_ values may be caused partly by a change in affinity of SR48692 to the NTSR1 mutants, and/or because SR48692 and [^3^H]NTS binding to the NTSR1 mutants did not reach equilibrium under the experimental conditions because of the change in the off-rate of [^3^H]NTS for the receptor mutants.

NTSR1-GW5, NTSR1-LF and NTSR1-ELF showed reduced sensitivity of agonist binding in the presence of Na^+^ ions (Supplementary Table 2) possibly indicating a high-affinity agonist conformation of the NTSR1 mutants. In contrast to wild-type NTSR1, the presence of NaCl did not increase the dissociation of [^3^H]NTS from NTSR1-LF, as was also observed with NTSR1-GW5 (ref. 3). However, NaCl did increase the dissociation of [^3^H]NTS from NTSR1-ELF, albeit not as pronounced as that seen with the wild-type receptor (Supplementary Fig. 1). The crystal structures of NTSR1-LF-T4L and NTSR1-ELF-T4L cannot explain the observed differences in the Na^+^ ion-dependent kinetics of NTS dissociation from NTSR1-LF and NTSR1-ELF. However, NTSR1-ELF may be more dynamic than NTSR1-LF, indicated by its lower thermal stability (Supplementary Table 3) and thus higher flexibility, accounting for the observed differences of the kinetic properties of both receptor mutants.

In G-protein-coupling assays, NTSR1-LF had moderate ability to activate the G protein. NTSR1-ELF was able to stimulate nucleotide exchange at Gαq to almost wild-type level (Fig. 1, Supplementary Fig. 1, Supplementary Table 2). The dose-response curves for the determination of the half maximal effective concentration of NTS on the exchange of GDP for GTPγS on Gq (EC_50_ values) were multiphasic (Hill slope <1) for NTSR1-ELF and wild-type NTSR1, indicating high and low affinity agonist-binding sites at non-saturating G-protein concentrations. In contrast, NTSR1-LF showed monophasic curves indicating a single class of binding sites. The EC_50_ value for wild-type NTSR1 is 20-fold higher than the K_i_ value determined in homologous [^3^H]NTS/NTS competition experiments, possibly because the affinity of NTS for wild-type NTSR1 is affected by the presence of Na^+^ ions (discussed above) reducing the efficacy of G-protein activation at the NaCl concentrations used in the nucleotide exchange reactions. The differences between EC_50_ and K_i_ values persist for NTSR1-LF and NTSR1-ELF, although they become smaller (15-fold and 4-fold for NTSR1-LF and NTSR1-ELF, respectively). As the NTSR1 mutants show reduced sensitivity of agonist binding in the presence of Na^+^ ions, the above explanation(s) may not suffice, but, in addition, possibly reflect the consequence of non-saturating G-protein concentrations in the GDP/GTPγS exchange assays (which may also apply to wild-type NTSR1).

### Architecture of NTSR1-ELF and NTSR1-LF

To understand the structural implications of E166^3.49^, L310^6.37^ and F358^7.42^ on the activation of G protein, we determined the structure of NTSR1-ELF to 2.9 Å resolution (Table 1). In addition, we determined the structure of NTSR1-LF (with L310 and F358) to 2.6 Å resolution. This latter mutant has moderate ability to activate G protein (Fig. 1) likely because of the absence of the E166^3.49^ side chain. The comparison with NTSR1-ELF is thus expected to provide insight into the structural role of E166^3.49^, a residue critical for governing receptor conformation and G-protein recognition^15^. For both constructs, we obtained crystals using the lipidic cubic phase crystallization method in combination with the chimeric T4 lysozyme approach to improve the probability of obtaining well-diffracting crystals.

**Figure 2:**
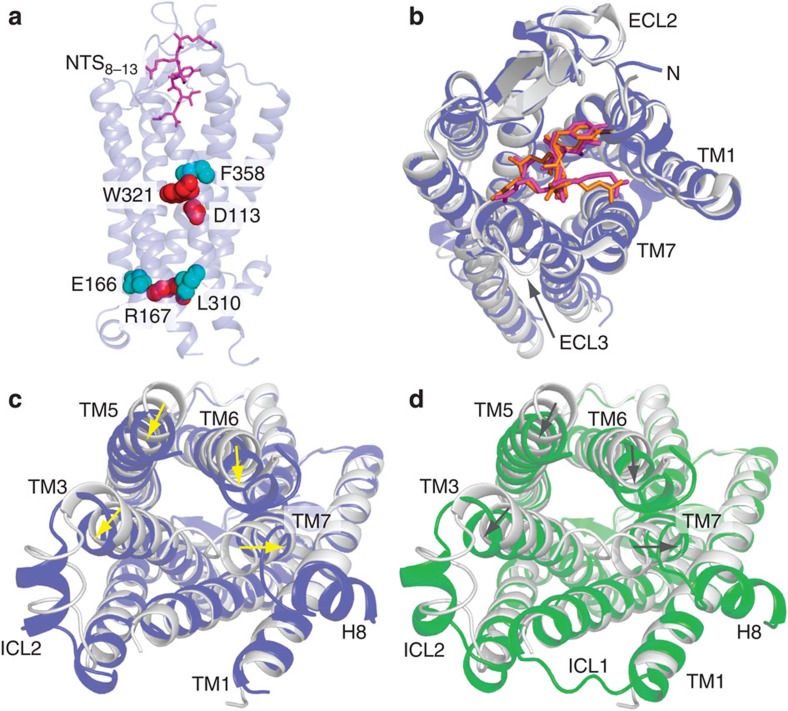
Overview of NTSR1 structures bound to the peptide agonist NTS_8–13_. Cartoon representation of NTSR1-ELF-T4L (blue; NTS_8–13_ in purple), NTSR1-LF-T4L (green) and NTSR1-GW5-T4L (grey, NTS_8–13_ in orange, PDB code 4GRV). NTS_8–13_ is depicted as a stick model. (**a**) Side view of NTSR1-ELF-T4L. Residues E166^3.49^, L310^6.37^ and F358^7.42^ are shown as cyan spheres; residues D113^2.50^, W321^6.48^ and R167^3.50^ are depicted in red. (**b**) Extracellular view. An arrow indicates ECL3, which is shifted towards the receptor core in NTSR1-ELF-T4L. (**c**,**d**) Intracellular view. Arrows indicate the position shift of the intracellular ends of TM3, TM5, TM6 and TM7 of NTSR1-ELF-T4L (**c**) and NTSR1-LF-T4L (**d**) compared with NTSR1-GW5-T4L. ICL1 is disordered in NTSR1-ELF-T4L. In contrast to NTSR1-GW5-T4L, NTSR1-ELF-T4L and NTSR1-LF-T4L have a short helix H8. T4L has been omitted from the intracellular view for clarity.

Superposition of NTSR1-ELF or NTSR1-LF with NTSR1-GW5 reveals that the structures are overall similar (root mean squared deviation with values of 0.7 Å for Cα atoms, excluding T4 lysozyme) indicating active-like NTSR1 conformations (Fig. 2). Despite the overall similar architecture of the three NTSR1 structures, NTSR1-ELF and NTSR1-LF differ from NTSR1-GW5 in a number of regions. The amino (N) terminus adopts a short helix (S53–L55) and is, compared with NTSR1-GW5, extended by two residues (G50 and P51) in NTSR1-ELF and three residues (A49, G50 and P51) in NTSR1-LF, providing additional contacts to extracellular loop (ECL) 2. ECL3 (residues I334-T340) of NTSR1-ELF and NTSR1-LF is shifted slightly towards the receptor core by ∼2.5 Å (Fig. 2). Subtle, yet distinct differences exist in the NTS_8–13_ binding mode (Supplementary Tables 4–7). Its R8 side chain is in strong hydrogen bond-mediated contact with D54 and D56 of the receptor N terminus. The R8 side chain in NTSR1-LF (but not in NTSR1-ELF) is also connected to TM7 through water-mediated hydrogen bonds to D345^7.29^. The R9 side chain also forms a strong hydrogen bond with the main chain oxygen of I334 of ECL3. Overall, NTS_8–13_ engages in more hydrogen bond-mediated interactions with NTSR1-LF and NTSR1-ELF compared with NTSR1-GW5 (Supplementary Tables 4–7).

On the intracellular side, the ends of TM3 of NTSR1-LF and NTSR1-ELF have shifted outward, whereas the ends of TM5 and TM6 have moved towards the receptor core (Fig. 2, Supplementary Table 8) compared with NTSR1-GW5. In addition, the relative positions between TMs within a given receptor have changed. TM3 and TM6 are positioned closer to each other, whereas TM5 and TM6 have shifted away from each other compared with NTSR1-GW5. Of the intracellular loops, ICL1 showed electron density for the main chain atoms in NTSR1-LF but not in NTSR1-ELF. ICL2, thought to be important for G-protein coupling, adopts a two-turn α helix in a slightly different position compared with the π helix seen in NTSR1-GW5 (Fig. 2). The structural significance of this observation for the ability of NTSR1 to activate G protein is unclear, as ICL2 adopts an α-helical structure in the active β_2_-adrenergic receptor–Gs complex^7^ and in the inactive β_1_AR structure^16^ but is an extended loop in the inactive β_2_AR structure^17^.

### Transmembrane helix 7 and helix 8

**Figure 3:**
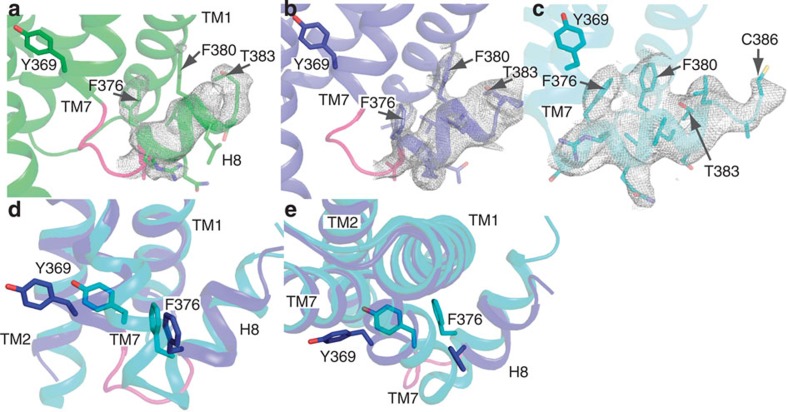
TM7 and helix 8. (**a**) NTSR1-LF-T4L is coloured green with mesh outlining the electron density of H8. (**b**) NTSR1-ELF-T4L in blue. (**c**) NTSR1 mutant TM86V-ΔIC3A in cyan (PDB code 3ZEV^18^). The partial unwinding of TM7 in NTSR1-ELF-T4L and NTSR1-LF-T4L (indicated by the pink colour) after the conserved NP^7.50^xxY motif allows F376^8.50^ to adopt its current position. The aromatic ring of F376^8.50^ is weakly anchored between TM1 and TM7 in NTSR1-LF-T4L, but not in NTSR1-ELF-T4L. The 2mFo-DFc sigma-A weighted maps are contoured at 1σ. (**d**,**e**) Comparison of active-like NTSR1-ELF-T4L (blue) with TM86V-ΔIC3A (cyan). TM3–TM6 have been omitted for clarity.

In contrast to NTSR1-GW5, TM7 shows partial ‘unwinding’ after the conserved NPxxY motif around N370^7.54^ (Fig. 3), similar to that seen in the structure of the active muscarinic acetylcholine M2 receptor^5^, but not in the structure of active β_2_AR^7^. This region (L371–N375) is followed by a short helix 8 (H8) in both NTSR1-LF and NTSR1-ELF structures. The aromatic ring of F376^8.50^, a conserved residue of the H8 motif, is well resolved in NTSR1-LF but is only weakly anchored in a hydrophobic pocket between TM1 and TM7. In NTSR1-ELF, F376^8.50^ is no longer anchored between TM1 and TM7 but is rotated outward forming hydrophobic interactions with L371. These features of NTSR1-LF and NTSR1-ELF are distinct from the NTSR1 mutant TM86V-ΔIC3A, which adopts an apparent inactive receptor conformation at the inner side in the crystal structure, for example, lacking the outward movement of TM6 (ref. 18). In TM86V-ΔIC3A, TM7 does not unwind as seen in NTSR1-ELF. The residue F376^8.50^ is partially inserted into the pocket between TM1, TM2 and TM7, whereas F376^8.50^ of NTSR1-ELF is not anchored into the receptor core (Fig. 3). In addition, H8 of NTSR1-ELF and NTSR1-LF is shorter than that of TM86V-ΔIC3A by two and three residues from the carboxy (C) terminus, respectively (Supplementary Table 9). Whether this reflects a general instability of H8 (ref. 18), or its dynamic nature in the active-like receptor conformation, remains to be explored. Recent molecular dynamics simulations suggested that unravelling of H8 is related to the agonist-occupied state of NTSR1 (ref. 19).

### The conserved residue W321^6.48^ of the CWxP motif

**Figure 4:**
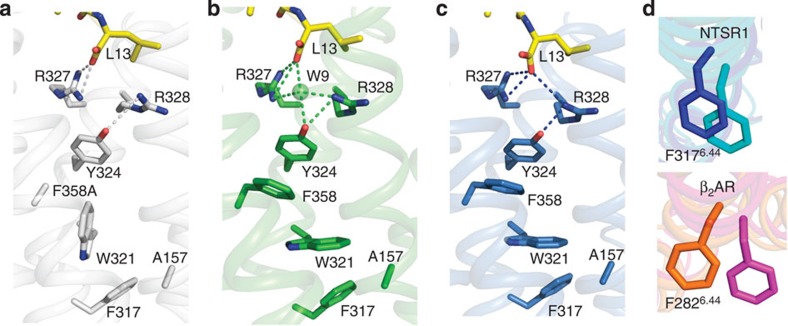
A hydrogen bond and van der Waals system links the agonist peptide with the hydrophobic core of NTSR1-LF and NTSR1-ELF. NTSR1-GW5-T4L (**a**) (PDB code 4GRV), NTSR1-LF-T4L (**b**) and NTSR1-ELF-T4L (**c**) are shown in grey, green and blue, respectively. Individual residues are shown as a stick model and are labelled. L13 of NTS_8–13_ (yellow) is connected to R327^6.54^, R328^6.55^ and Y324^6.51^ via a hydrogen bond network. Water molecule W9 is labelled. A close-by water molecule W10 that is bonded to L13 of NTS_8–13_ in NTSR1-LF-T4L has been omitted for clarity. No corresponding water molecules were detected in NTSR1-ELF-T4L, likely because of the lower resolution of the structure. A series of stacking interactions relate Y324^6.51^ to F358^7.42^, W321^6.48^ and the hydrophobic core^23^ residue F317^6.44^. (**d**) Position of the F^6.44^ side chain in NTSR1-ELF-T4L (blue), the NTSR1 mutant TM86V-ΔIC3A (cyan, PDB code 3ZEV) and the inactive (purple, PDB code 2RH1) and active β_2_AR (orange, PDB code 3SN6), viewed from the intracellular side.

Of significance is the position of W321^6.48^ within the CWxP motif, a highly conserved amino acid in class A GPCRs. Spectroscopic evidence suggested changes in the environment of W^6.48^ upon rhodopsin activation^20^,^21^. However, a rotamer change of W^6.48^ is not observed in any crystal structure of active rhodopsin^22^, the β_2_AR^6^ or the M2 receptor^5^ suggesting that changes in the W^6.48^ rotamer orientation might not be an essential part of the GPCR activation mechanism^6^. In contrast to all the previously determined GPCR structures, the W321^6.48^ side chain in NTSR1-LF and NTSR1-ELF is oriented parallel to the lipid bilayer (Fig. 4; Supplementary Fig. 2). This orientation results from the presence of F358^7.42^ whose phenyl side chain prevents W321^6.48^ from adopting the side chain conformation found in NTSR1-GW5, which contained the stabilizing F358A^7.42^ mutation. Consequently, the W321^6.48^ indole side chain makes additional van der Waals interactions with residues of TM3, TM5 and TM6 (Supplementary Fig. 3). It is worth noting that 73% of class A GPCRs, including rhodopsin, have small residues (G, A, S) at position 7.42; bulky tyrosine and phenylalanine residues are rare and comprise only 4% of class A GPCRs. For example, neuromedin U receptors and NTSR2 have a phenylalanine at position 7.42 and a tryptophan residue at position 6.48. The luteinizing hormone/choriogonadotropin and thyroid-stimulating hormone receptors have a tyrosine at position 7.42, but a methionine at position 6.48 *in lieu* of a tryptophan residue. All class A receptors, for which crystal structures have been determined to date, have a small residue at position 7.42 (muscarinic receptors have a cysteine^7.42^ residue, the P2Y_12_ receptor has a threonine^7.42^ residue, the orexin OX2 receptor has a valine^7.42^ residue) except NTSR1, possibly explaining in part why the W^6.48^ rotamer conformation seen here in NTSR1-LF and NTSR1-ELF has not been observed in other receptor structures.

### A network of interactions links NTS to the hydrophobic core

NTSR1-LF and NTSR1-ELF structures help explain how the agonist peptide transmits its extracellular signal to the intracellular portion of the receptor. Hydrogen bond and van der Waals interactions link NTS_8–13_ with residues of the hydrophobic core associated with helical rearrangements seen in active-state structures^23^ (Fig. 4). NTS_8–13_ is connected to Y324^6.51^ via a hydrogen bond network from the carboxylate of its L13 residue through R328^6.55^. The aromatic ring of Y324^6.51^, in turn, is engaged in hydrophobic stacking interactions with F358^7.42^ that is in contact with W321^6.48^, as previously discussed. The hydrophobic network results in the packing of W321^6.48^ against the hydrophobic F317^6.44^ that has been implicated in the reorganization of transmembrane segments upon agonist binding in β_2_AR^6^. The rotamer position of F317^6.44^ is almost the same in NTSR1-GW5, NTSR1-LF and NTSR1-ELF (Fig. 4a–c). This is perhaps not surprising as all the three structures represent active-like receptor states (the differences in the ability to activate G protein may, in part, be attributed to the positioning of R167^3.50^ by L310^6.37^, and the presence of E166^3.49^, as discussed). However, comparison of NTSR1-ELF with the mutant TM86V-ΔIC3A^18^, which represents an inactive NTSR1 at the inner side, suggests a rearrangement of the F317^6.44^ side chain (Fig. 4d). The extent of the F317^6.44^ repacking upon activation of NTSR1 remains to be determined as F317^6.44^ in TM86V-ΔIC3A is in a receptor region, which may not represent a fully inactive conformation. The network of hydrophobic interactions identified in NTSR1-LF and NTSR1-ELF were not seen in the NTSR1-GW5 structure owing to the F358A^7.42^ stabilizing mutation.

### The collapsed Na^+^ ion-binding pocket

**Figure 5:**
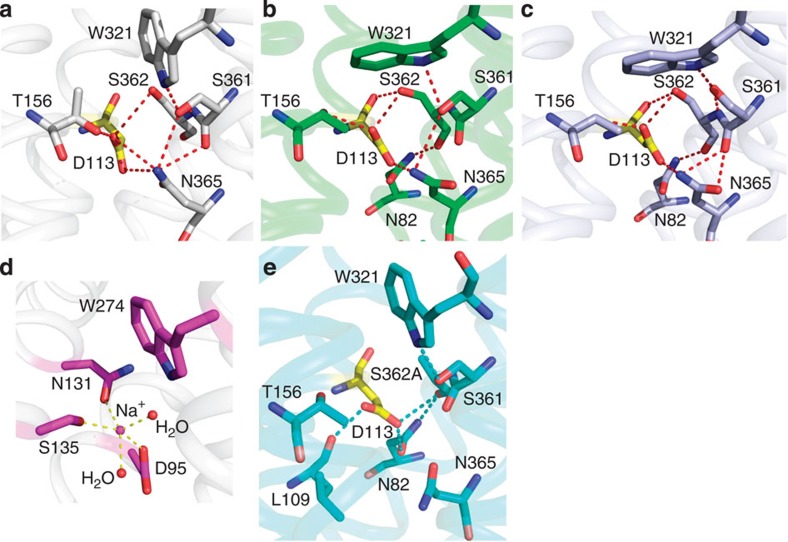
The collapsed Na^+^ ion-binding pocket. (**a**) NTSR1-GW5-T4L (grey, PDB code 4GRV), (**b**) NTSR1-LF-T4L (green), (**c**) NTSR1-ELF-T4L (blue). The conserved D113^2.50^ residue (yellow), which has been assigned a crucial role in the Na^+^ ion sensitivity of agonist binding, engages in hydrogen bond interactions with T156^3.39^, S362^7.46^ and N365^7.49^ preventing the coordination of a Na^+^ ion. Other polar interactions between nearby residues are also shown. Residues of TM5 have been removed from the cartoons for clarity. (**d**) Inactive δ-opioid receptor (purple, PDB code 4N6H^25^) with a Na^+^ ion coordinated by residues D95^2.50^, N131^3.35^ S135^3.39^ and two water molecules. (**e**) NTSR1 mutant TM86V-ΔIC3A (cyan, PDB code 3ZEV). Polar interactions are shown as dashed cyan lines. No electron density for water molecules or a Na^+^ ion has been reported^18^.

Na^+^ ions have a negative allosteric effect on agonist binding to wild-type NTSR1 and the highly conserved D113^2.50^ in the middle of TM2 has been assigned a pivotal role in the Na^+^ ion sensitivity of agonist binding and G-protein activation^24^. Recent high-resolution structures of GPCRs in the inactive state have revealed a conserved Na^+^ ion-binding pocket within the receptor transmembrane bundle^25^,^26^,^27^,^28^, providing a structural explanation for the allosteric effect of Na^+^ ions on agonist binding. In each of those inactive structures, the Na^+^ ion is coordinated by a salt bridge to the highly conserved D^2.50^ and by four additional contacts with receptor side chains and water molecules (Fig. 5d). In the active-like NTSR1-LF and NTSR1-ELF structures (and in NTSR1-GW5), the Na^+^ ion-binding pocket has collapsed (Fig. 5), which explains the reduced Na^+^ ion sensitivity of agonist binding (Supplementary Table 2). The D113^2.50^ side chain atoms form an extensive hydrogen bond network with T156^3.39^, S362^7.46^ and N365^7.49^ of the NPxxY motif, preventing the coordination of a Na^+^ ion. Absent in the collapsed NTSR1 Na^+^ ion pocket are any water molecules, which fill the cavity in inactive-state receptors^26^. In NTSR1-LF and NTSR1-ELF, W321^6.48^ forms van der Waals interaction with residues of the Na^+^ ion-binding pocket, effectively sealing off the top of the collapsed Na^+^ ion pocket and disrupting a vertical cavity seen in NTSR1-GW5 (Supplementary Fig. 4).

The structure of the NTSR1 mutant TM86V-ΔIC3A^18^ is similar to our active-like NTSR1 structures in the extracellular half, which is responsible for ligand binding, but dissimilar in the intracellular half, adopting an apparent inactive receptor conformation in the crystal structure. The Na^+^ ion-binding region is located underneath the ligand binding pocket between the intracellular and extracellular receptor halves. In TM86V-ΔIC3A, the D113^2.50^ side chain is also in contact with neighbouring residues, albeit the interactions are different when compared with NTSR1-GW5, NTSR1-LF and NTSR1-ELF (Fig. 5). In addition, no electron density for water molecules or a Na^+^ ion has been reported in the TM86V-ΔIC3A structure^18^. Note that S362^7.46^, which contacts D113^2.50^ in NTSR1-LF and NTSR1-ELF, is mutated to an alanine residue in TM86V-ΔIC3A (Fig. 5).

### The residue L310^6.37^ positions the R167^3.50^ side chain

**Figure 6:**
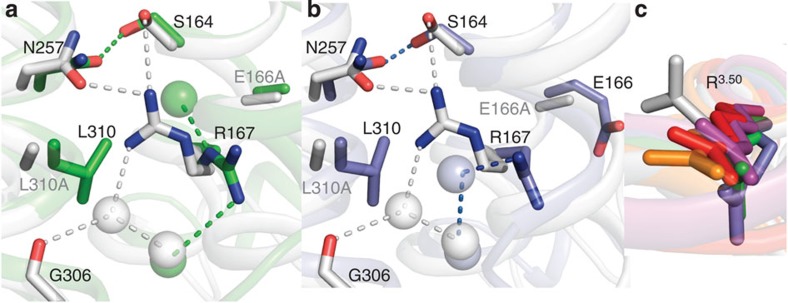
Effect of L310^6.37^ on the positioning of the R167^3.50^ side chain. (**a**,**b**) Comparison of NTSR1-LF-T4L (green) and NTSR1-ELF-T4L (blue) with NTSR1-GW5-T4L (grey, PDB code 4GRV). Hydrogen bonds are indicated by dashed lines, and water molecules are represented as spheres. In NTSR1-GW5-T4L, the R167^3.50^ side chain is stabilized by a hydrogen bond network to N257^5.58^, S164^3.47^ and G306^6.33^, facilitated by the L310A mutation. The presence of the larger side chain of L310^6.37^ in NTSR1-LF-T4L and NTSR1-ELF-T4L is sterically incompatible with such an arrangement and the R167^3.50^ side chain interactions with N257^5.58^ and S164^3.47^ are lost. (**c**) The R167 side chain now adopts a conformation similar to that seen in metarhodopsin II (ref. 4; purple, PDB code 3PQR), the β_2_AR–Gs complex^7^ (orange, PDB code 3SN6) and the active M2 receptor^5^ (red, PDB code 4MQS).

The residue at position 6.37 (L310^6.37^ in NTSR1) is highly conserved among class A GPCRs; 80% of receptors have hydrophobic residues (L, I, V) at this position. The significance of this residue becomes apparent in the NTSR1-LF and NTSR1-ELF structures (Fig. 6). L310^6.37^ is central to the positioning of the R167^3.50^ side chain such as to allow a bona fide productive interaction with the G protein. In the signalling incompetent, active-like NTSR1-GW5 structure, R167^3.50^ is linked to the conserved N257^5.58^, S164^3.47^ and G306^6.33^ by a hydrogen bond network, likely facilitated by the decreased side chain size of the L310A^6.37^ stabilizing mutation. Those interactions stabilize R167^3.50^ in a position unfavourable for contacting the Gα subunit. The presence of the larger L310^6.37^ side chain prevents R167^3.50^ from adopting the conformation seen in NTSR1-GW5 (Fig. 6). In NTSR1-LF and NTSR1-ELF, the R167^3.50^ side chain has an orientation that is similar to that found in the β_2_AR–Gs complex^7^, the active M2 receptor^5^ and opsin in its G-protein-interacting conformation^29^. The current NTSR1 structures represent snapshots of an active-like receptor bound to agonist, but not to a G protein, possibly explaining subtle differences of the R167^3.50^ side-chain conformations compared with receptor structures in complex with G protein or G protein mimetics (Fig. 6).

## Discussion

Stabilization of detergent-solubilized receptor–ligand complexes is one of the key factors for successful crystallization and structure determination of membrane proteins. Wild-type NTSR1 is not particularly stable in detergent solution^30^; thus the use of stabilized NTSR1 mutants has resulted in the successful production of well-diffracting crystals (Supplementary Table 3). Our previously reported active-like NTSR1-GW5 mutant^3^ was obtained by conformational thermostabilization^31^ in the presence of the agonist neurotensin^12^,^30^. An alternative approach, directed evolution^32^,^33^,^34^, has resulted in structures of NTSR1 (ref. 18), which are similar to our active-like NTSR1 structures in the extracellular half but have adopted an apparent inactive receptor conformation in the intracellular half.

The structures of NTSR1-LF and NTSR1-ELF, presented here, are very similar (root mean squared deviation values of 0.3 Å for Cα atoms, excluding T4 lysozyme), yet their ability to activate the G protein in response to NTS differs: NTSR1-LF mediates moderate nucleotide exchange at Gαq, whereas NTSR1-ELF has almost wild-type receptor properties (Supplementary Table 2). The glutamic acid^3.49^ of the highly conserved D/ERY motif, absent in NTSR1-LF, but present in NTSR1-ELF, has been deemed critical for G-protein coupling^15^. Thus the mutation E166A^3.49^ alone may explain the pharmacological behaviour of NTSR1-LF, highlighting the importance of E166^3.49^ for G-protein activation. In the active M2 receptor, D120^3.49^ is stabilized by a hydrogen bond with N58^2.39^ (T68^2.39^ in β_2_AR); and it has been suggested that N58^2.39^ either directly stabilizes the active receptor conformation, or engages in direct interactions with G protein^5^. The equivalent residue in NTSR1 is V102^2.39^ precluding side chain hydrogen bond interactions; instead, E166^3.49^ is hydrogen bonded to the side chain of T101^2.38^, the main chain amide of V102^2.39^, and weakly linked to H105^2.42^ (Supplementary Fig. 5). In the β_2_AR–Gs complex^7^, T68^2.39^ and D130^3.49^ interact with the ICL2 helix via Y141^ICL2^ positioning the helix such that a phenylalanine docks into a hydrophobic pocket on the G protein surface. ICL2 has been found essential for the G-protein activation pathway, especially for the dissociation of the receptor–G protein complex in the presence of GTP^35^. Thus E166^3.49^ may optimally position ICL2 in the presence of G protein allowing efficient G-protein binding and release. In β_2_AR, Y141^ICL2^ links the receptor–G protein interactions of ICL2 with the D/ERY motif. The equivalent interaction in NTSR1 may come by M181^ICL2^ as the NTSR1 residue, equivalent to Y141^ICL2^ of β_2_AR, is an alanine (A177).

In conclusion, our current NTSR1 structures provide insight into mechanistic details of an active-like, agonist-occupied peptide GPCR. The conserved W321^6.48^, oriented parallel to the membrane plane, seals the top of a collapsed Na^+^ ion-binding pocket; W321^6.48^ in combination with F358^7.42^ link the agonist peptide, bound near the receptor surface, with hydrophobic core residues in the inner half of NTSR1. The highly conserved residue L310^6.37^ in the vicinity of the D/ERY motif is central to the side-chain orientation of R167^3.50^ to promote the productive interaction with Gq protein. The neighbouring E166^3.49^ residue is vital for G-protein activation, possibly by coupling receptor–G protein interactions with the D/ERY motif.

## Methods

### NTSR1 constructs

The baculovirus construct NTSR1-LF-T4L consisted of the hemagglutinin signal peptide and the Flag tag^36^, followed by the thermostabilized rat NTSR1 (T43-K396 containing the mutations A86L, E166A, G215A, V360A) with the ICL3 residues H269-E296 replaced by the cysteine-free bacteriophage T4 lysozyme (N2-Y161 with the mutations C54T and C97A) and a GSGS linker. A deca-histidine tag was placed at the C terminus. NTSR1-LF contained the wild-type ICL3 sequence. NTSR1-ELF-T4L and NTSR1-ELF were like NTSR1-LF-T4L and NTSR1-LF, respectively, but had only three mutations (A86L, G215A, V360A). The wild-type NTSR1 used here was like NTSR1-LF but did not have the four mutations. Additional NTSR1 mutants, used for pharmacological analyses, are listed in Supplementary Table 1.

In the Methods sections, we distinguish between NTSR1 constructs containing T4L or the wild-type ICL3 sequence. In the main text, we use only one name for a particular construct; for example, NTSR1-ELF refers interchangeably to NTSR1-ELF-T4L and NTSR1-ELF, the latter containing the wild-type ICL3, not T4L. The identity of the respective construct is evident from the context of writing.

### Expression of NTSR1 in insect cells

Recombinant baculoviruses were generated using a modified pFastBac1 transfer plasmid (Invitrogen). *Trichoplusia ni* cells were infected with recombinant virus, and the temperature was lowered from 27 to 21 °C. Cells were collected by centrifugation 48 h post infection, resuspended in hypotonic buffer (10 mM Hepes pH 7.5, 10 mM MgCl_2_, 20 mM KCl), flash frozen in liquid nitrogen and stored at −80 °C until use.

### Expression of Gq protein in insect cells

The baculovirus construct His-Tev-Gαq consisted of a hexa-histidine tag, followed by a tobacco etch virus (TEV) protease recognition site and the human Gαq sequence (M7-V359). Human Gβ_1_ was unmodified. Human Gγ_1_ was preceded by a hexa-histidine tag. We refer to His-Tev-Gαq Gβ_1_ His-Gγ_1_ as Gq protein. *Trichoplusia ni* cells were triple-infected with the recombinant viruses at 27 °C. Cells were collected by centrifugation 48 h post infection, resuspended in hypotonic buffer (10 mM Hepes pH 7.5, 10 mM MgCl_2_, 20 mM KCl), flash frozen in liquid nitrogen and stored at −80 °C until use.

### Preparation of urea-washed P2 insect cell membranes

NTSR1-enriched membranes were obtained as a P2 fraction from insect cells^30^,^37^. Before G-protein-coupling assays and ligand binding experiments, the P2 membranes were treated with urea to remove peripherally bound membrane proteins^38^,^39^. The receptor density in urea-washed P2 membranes was determined by [^3^H]NTS saturation binding analysis^30^.

### Ligand-binding experiments

All radioligand binding assays were conducted with urea-washed P2 insect cell membranes containing the indicated NTSR1 constructs. Independent experiments were carried out in single data points.

For agonist [^3^H]NTS ([3,11-tyrosyl-3,5-3H(N)]-pyroGlu-Leu-Tyr-Glu-Asn-Lys-Pro-Arg-Arg-Pro-Tyr-Ile-Leu; PerkinElmer) saturation binding experiments, receptors were incubated on ice for 1 h in 250 μl TEBB buffer (50 mM TrisHCl pH 7.4, 1 mM ethylenediaminetetraacetic acid (EDTA), 40 μg ml^−1^ bacitracin, 0.1% (w/v) BSA) containing [^3^H]NTS at a concentration of 0.6–20 nM. Nonspecific [^3^H]NTS binding was determined in the presence of 50 μM unlabelled NTS. Separation of bound from free ligand was achieved by rapid filtration through GF/B glass fibre filters (Whatman) pretreated with polyethylenimine (0.5% w/v). The amount of radioactivity was quantified by liquid scintillation counting (Beckman LS 6500). Data were analysed by nonlinear regression using the GraphPad Prism software and best fit to a one-site binding equation to determine the dissociation constants (K_d_). Note that the saturation binding experiments using wild-type NTSR1 were conducted at equilibrium. In contrast, binding of [^3^H]NTS to the NTSR1 mutants did not reach equilibrium within the incubation time because of the slow agonist off-rates (discussed previously).

Homologous competition assays with NTS were performed in the presence of [^3^H]NTS (TEBB buffer, 4.5–5 nM [^3^H]NTS, NTSR1 concentration <0.5 nM, incubation for 2 h on ice, 250 μl assay volume). Data were best fit to a sigmoidal dose-response equation with standard slope using the concentrations of total NTS added versus bound [^3^H]NTS. Inhibition constant (K_i_) values were derived from IC_50_ values using the Cheng and Prusoff equation, K_i_=IC_50_/(1+L/K_d_), where L is the concentration of [^3^H]NTS (ref. 40).

Competition assays with the nonpeptide antagonist SR48692 (ref. 14) were performed in the presence of [^3^H]NTS (TEBB buffer, 5 nM [^3^H]NTS, NTSR1 concentration <0.5 nM, incubation for 2 h on ice, 250 μl assay volume). Data were analysed by nonlinear regression with the GraphPad Prism three-parameter dose-response equation (standard slope) using the concentrations of total SR48692 added versus bound [^3^H]NTS.

The effect of Na^+^ ions on [^3^H]NTS binding was measured with NaCl concentrations ranging from 0 to 2 M (TEBB buffer, 8 nM [^3^H]NTS, NTSR1 concentration <0.5 nM, incubation for 1.5 h on ice, 300 μl assay volume). Data were analysed by nonlinear regression using the GraphPad Prism four-parameter dose-response equation (variable slope) with the top and bottom plateaux constrained from 100% to 10% (wild-type NTSR1) and 100% to 50% (NTSR1-LF-T4L, NTSR1-LF, NTSR1-ELF-T4L, NTSR1-ELF), respectively.

The association of [^3^H]NTS was assessed at a concentration of 10 nM (TEBB buffer, NTSR1 concentration <0.5 nM). At the indicated time points, 250 μl aliquots were filtered over glass fibre filters. After 2 h, [^3^H]NTS dissociation was initiated by adding 41.7 μM unlabelled NTS or by the addition of 41.7 μM NTS and 833 mM NaCl; this step reduced the concentration of [^3^H]NTS to 8 nM. The samples were subjected to filtration after the indicated time points. No attempt was made to quantitatively compare the observed rates of association and the dissociation rate constants between NTSR1 constructs because of the very fast association of agonist to wild-type NTSR1 (ref. 3), the very fast dissociation of agonist from wild-type NTSR1 in the presence of NaCl (ref. 3) and the slow dissociation of [^3^H]NTS from the NTSR1 mutants.

### GTPγS assays

Before G-protein-coupling assays, the P2 membranes were treated with urea to remove peripherally bound membrane proteins^38^,^39^. GDP/[^35^S]GTPγS exchange assays were performed^30^,^41^ with 1 nM receptor, 140 nM Gq protein and specified amounts of NTS, nonpeptide antagonist SR48692 (ref. 14) or no ligand in the reaction. Experiments were conducted either at saturating ligand concentrations (NTS at 20 μM, SR48692 at 40 μM) or using a range of ligand concentrations for dose-response assays (0–20 μM NTS, 0–40 μM SR48692). Independent experiments were carried out in single data points. Urea-washed membranes containing NTSR1 were added to Gq protein and ligand to give a volume of 30 μl. GDP/GTPγS exchange was initiated by the addition of 20 μl of [^35^S]GTPγS mix (5 min at 30 °C). The final component concentrations in each sample were 50 mM MOPS (3-(N-morpholino)propanesulfonic acid) (pH 7.5), 1 mM EDTA, 120 mM NaCl, 1 mM dithiothreitol (DTT), 3 mM MgSO_4_, 0.3% (w/v) BSA, 1 μM GDP, 4–8 nM [^35^S]GTPγS (PerkinElmer), 40 μM adenylyl imidodiphosphate, 0.4 mM cytidine 5′-monophosphate, 0.1% (w/v) 3-[(3-cholamidopropyl)dimethylammonio]propanesulfonic acid (CHAPS; Anatrace). Reactions were terminated by diluting the reaction mixture with 2 ml of ice-cold stop buffer (20 mM TrisHCl pH 8.0, 100 mM NaCl and 25 mM MgCl_2_) and were filtered over nitrocellulose membranes on a vacuum manifold. Filters were then washed six times with 2 ml of ice-cold stop buffer. The nitrocellulose membranes were dried, and the radioactivity was quantified by liquid scintillation in a Beckman Coulter LS 6500 scintillation counter. Data from dose-response experiments were fit to equations with a Hill slope of 1 or variable slope.

The Gq protein used for exchange assays was purified in buffer containing CHAPS. However, the Gq protein addition in the exchange assays did not exceed one-fifth of the reaction volume, thus limiting the free CHAPS concentration to one-fourth of its critical micellar concentration^42^.

The quantification of purified Gq protein was done as above with the following modifications. Duplicate reaction mixes (final volume of 75 μl) contained 1 μM unlabelled GTPγS instead of GDP, 25 nM receptor, a defined amount of the Gq protein preparation and 10 μM NTS or no ligand. Aliquots (10 μl) of the reaction mixes were transferred into 2 ml of ice-cold stop buffer after 5, 10, 20 and 30 min. [^35^S]GTP**γ**S binding in the absence of NTS (non-catalysed nucleotide exchange) was subtracted from [^35^S]GTP**γ**S binding in the presence of NTS (total nucleotide exchange). The resulting data were fit to a one-site binding model in the Prism software (GraphPad) to calculate the concentration of His-Tev-Gαq.

### Purification of Gq protein from insect cells

All the steps were performed at 4 °C or on ice. Cells from 2 to 4 l of insect cell culture were thawed and sedimented by centrifugation (45Ti rotor, 125,000*g*, 25 min, 4 °C, Optima L90K, Beckman). The pellet was resuspended in buffer using a glass (dounce) tissue grinder with tight pestle. After drop-wise addition of a 10% (w/v) CHAPS solution, the sample was stirred for 1.5–2 h. The final volume was 50 ml per liter of cell culture, containing 20 mM Hepes pH 8.0, 300 mM NaCl, 5 mM MgCl_2_, 5 mM β-mercaptoethanol, 10 μM GDP, 100 μM AEBSF (4-(2-Aminoethyl)benzene sulfonyl fluoride hydrochloride), 3 μg ml^−1^ leupeptin, 3 μg ml^−1^ trypsin inhibitor, 20 μg ml^−1^ tosyl lysyl chloromethyl ketone, 20 μg ml^−1^ tosyl phenylalanyl chloromethyl ketone, 50 μg ml^−1^ deoxyribonuclease, 1% (w/v) CHAPS. The sample was clarified by centrifugation (45Ti rotor, 125,000*g*, 1 h, Optima L90K, Beckman), diluted 1.5-fold with detergent-free buffer to reduce the CHAPS concentration to 0.67% (w/v), adjusted with imidazole to a final concentration of 50 mM, and batch-incubated overnight with Ni-NTA resin (1 ml resin per liter of cell culture) equilibrated with buffer GA (20 mM Hepes pH 8.0, 300 mM NaCl, 5 mM MgCl_2_, 5 mM β-mercaptoethanol, 10 μM GDP, 50 mM imidazole, 0.5% (w/v) CHAPS). After washing the resin with 15 column volumes of buffer GA, the G protein was eluted with buffer GB (20 mM Hepes pH 8.0, 300 mM NaCl, 5 mM MgCl_2_, 5 mM β-mercaptoethanol, 250 mM imidazole, 0.5% (w/v) CHAPS).

For some Gq protein preparations, the sample was subjected to ion exchange chromatography before the final size exclusion chromatography step. For this, protein containing Ni-NTA fractions were pooled, supplemented with 10 μM GDP, concentrated using a 30,000 MWCO concentrator (Amicon Ultra, Millipore) and passed over PD10 columns (GE Healthcare) equilibrated in IEX binding buffer (20 mM Hepes pH 8.0, 5 mM MgCl_2_, 1 mM DTT, 0.5% (w/v) CHAPS) supplemented with 10 μM GDP. Then the sample was concentrated and loaded onto a 1 ml HiTrap DEAE FF column (GE Healthcare) equilibrated in IEX binding buffer. After washing with IEX binding buffer, the Gq protein was eluted using a gradient to 100% elution buffer (20 mM Hepes pH 8.0, 5 mM MgCl_2_, 1 mM DTT, 500 mM NaCl, 0.5% (w/v) CHAPS) over 10 column volumes.

Finally, the protein containing Ni-NTA or IEX fractions were pooled and supplemented with 10 μM GDP, concentrated using a 30,000 MWCO concentrator (Amicon Ultra, Millipore) and run on a Superose 6 10/300 column (GE Healthcare) equilibrated in GF buffer (10 mM MOPS pH 7.5, 100 mM NaCl, 1 mM DTT, 0.5% (w/v) CHAPS). The fractions containing Gq protein were pooled, supplemented with 10 μM GDP, aliquoted, flash frozen and stored at −80 °C until use for pharmacological experiments.

### Purification of NTSR1-LF-T4L and NTSR1-ELF-T4L

The cells from 3 l of insect cell culture were thawed and the volume was brought to ∼240 ml with hypotonic buffer (10 mM Hepes pH 7.5, 10 mM MgCl_2_, 20 mM KCl). The cells were then resuspended using a Turrax T-25 (IKA) homogenizer at 8,000 r.p.m. for 2 min. After centrifugation (45Ti rotor, 125,000*g*, 20 min, 4 °C, Optima L90K, Beckman), the membranes were resuspended (Turrax T-25) in ∼180 ml of high-salt buffer (10 mM Hepes pH 7.5, 1 M NaCl, 10 mM MgCl_2_, 20 mM KCl) supplemented with AEBSF (100 μM) and centrifuged again. The high-salt buffer wash was repeated one more time resulting in ∼12 g of wet membrane pellet. All the subsequent steps were performed at 4 °C or on ice, and AEBSF (100 μM final concentration) was repeatedly added throughout the procedure. The washed membranes were resuspended in 122 ml of buffer (80 mM TrisHCl pH 7.4, 48% (v/v) glycerol) containing 16 μM NTS_8–13_ (Arg-Arg-Pro-Tyr-Ile-Leu) and stirred for 45 min to allow agonist binding to membrane-inserted NTSR1. The receptor was extracted by drop-wise addition of 65 ml of a 3% (w/v) lauryl maltose neopentyl glycol (2,2-didecylpropane-1,3-bis-β-D-maltopyranoside; LMNG; Anatrace)^43^/0.3% (w/v) CHS (cholesteryl hemisuccinate Tris salt) solution (Anatrace). After 1 h, NaCl was added and the solution was gently stirred for an additional 15 min. The final volume was 195 ml containing 50 mM TrisHCl pH 7.4, 30% (v/v) glycerol, 200 mM NaCl, 1% (w/v) LMNG/0.1% (w/v) CHS and 10 μM NTS_8–13_. The sample was clarified by centrifugation (45Ti rotor, 125,000*g*, 1 h, Optima L90K, Beckman), adjusted with imidazole to a final concentration of 20 mM and batch-incubated overnight with 1.5 ml Talon resin equilibrated with Talon-A^+^ buffer (50 mM TrisHCl pH 7.4, 30% (v/v) glycerol, 200 mM NaCl, 20 mM imidazole, 1 μM NTS_8–13_, 0.1% (w/v) LMNG/0.01% (w/v) CHS). After washing the resin with 22.5 ml of buffer Talon-A^+^ and 15 ml of buffer Talon-A2^+^ (50 mM TrisHCl pH 7.4, 30% (v/v) glycerol, 200 mM NaCl, 20 mM imidazole, 1 μM NTS_8–13_, 0.05% (w/v) LMNG/0.005% (w/v) CHS), NTSR1-LF-T4L and NTSR1-ELF-T4L were eluted in 0.5 ml steps with Talon-B^+^ buffer (50 mM TrisHCl pH 7.4, 30% (v/v) glycerol, 200 mM NaCl, 250 mM imidazole, 10 μM NTS_8–13_, 0.05% (w/v) LMNG/0.005% (w/v) CHS). Peak fractions were collected (2.5 ml) and desalted using a PD10 column equilibrated in PD10 buffer (50 mM TrisHCl pH 7.4, 200 mM NaCl, 0.003% (w/v) LMNG/0.0003% (w/v) CHS). NTS_8–13_ was then added to a concentration of 20 μM, and the sample was used for crystallization. Three litres of insect cell culture yielded ∼2 mg of purified NTSR1-LF-T4L or NTSR1-ELF-T4L.

### Stability tests in detergent solution

The cell pellets from 10 ml of insect cell cultures were resuspended in 1.8 ml buffer containing LMNG/CHS to give a final buffer composition of 50 mM TrisHCl pH 7.4, 200 mM NaCl, 1% (w/v) LMNG/0.1% (w/v) CHS. The samples were placed on a rotating mixer at 4 °C for 1 h. Cell debris and non-solubilized material were removed by ultracentrifugation (TLA 120.2 rotor, 128,000*g*, 4 °C, 30 min in Optima Max bench-top ultracentrifuge, Beckman) and the supernatants containing detergent-solubilized NTSR1 were used to test for thermal stability in the +NTS format^30^. For thermal denaturation curves, the supernatants were diluted 6.67-fold into assay buffer (50 mM TrisHCl pH 7.4, 200 mM NaCl) containing 10 nM [^3^H]NTS and incubated for 1–2.5 h on ice to allow [^3^H]NTS binding to NTSR1. The samples (120 μl aliquots) were exposed to different temperatures between 0 and 70 °C for 30 min and placed on ice. Separation of receptor–ligand complex from free ligand (100 μl) was achieved by centrifugation-assisted gel filtration (spin assay) using Bio-Spin 30 Tris columns (Bio-Rad), equilibrated with RDB buffer (50 mM TrisHCl pH 7.4, 1 mM EDTA, 0.1% (w/v) DDM (n-dodecyl-β-D-maltopyranoside) (Anatrace), 0.2% (w/v) CHAPS, 0.04% (w/v) CHS). Control reactions on ice were recorded at the start and at the end of each denaturation experiment. The percentage of activity remaining after heat exposure was determined with respect to the unheated control. Data were analysed by nonlinear regression using a Boltzmann sigmoidal equation in the Prism software (GraphPad).

### Crystallization

Purified desalted NTSR1-LF-T4L and NTSR1-ELF-T4L were adjusted to 100 μM Tris (2-carboxyethyl) phosphine hydrochloride (TCEP) and 350 μM NTS_8–13_ and concentrated to an estimated 30 mg ml^−1^ using a 100,000 MWCO concentrator (Amicon Ultra, Millipore). After the addition of NTS_8–13_ to 1.5 mM and centrifugation (TLA 120.1 rotor, 128,000*g*, 30 min, 4 °C, Beckman), the sample was mixed with 1.5 parts by weight of a mix of monoolein with cholesterol (10:1) using the two-syringe method^44^. The resulting lipidic cubic phase^45^ mix was dispensed in 65–75 nl drops onto Laminex plates (Molecular Dimensions) and overlaid with 750 nl (NTSR1-LF-T4L) or 875 nl (NTSR1-ELF-T4L) precipitant solution using a Mosquito LCP robot (TTP Labtech). Crystals of NTSR1-LF-T4L grew at 20 °C after 3 days in precipitant solution consisting of 19.8–23.4% (v/v) PEG 400, 80 mM Hepes pH 7.0–7.4, 2 mM TCEP and 50 mM lithium citrate. Crystals of NTSR1-ELF-T4L grew in precipitant solution consisting of 16–24% (v/v) PEG 400, 75 mM Hepes pH 7.0–8.0, 1.7 mM TCEP, 32 mM lithium citrate and 0.9 mM NTS_8–13_. Crystals were harvested directly from LCP using micro-loops (MiTeGen) and immediately flash frozen in liquid nitrogen without adding extra cryoprotectant.

### Data collection and structure determination

Data collection was performed using the JBluIce-EPICS data acquisition software at the GM/CA-CAT (23-ID-D) beamline at the Advanced Photon Source of the Argonne National Laboratory using a 10–20 μm minibeam at a wavelength of 1.0332 Å and a Pilatus 3 6 M detector. Crystals within the loops were located by diffraction using the automated rastering module of JBluIce-EPICS^46^,^47^. Partial data sets (wedges of 20–30 degrees) were collected from crystals exposed to the non-attenuated minibeam for 0.3 s and 0.3-degree oscillation per exposure.

For NTSR1-LF-T4L, a 99.4% complete data set at 2.6 Å resolution was obtained by indexing, integrating, scaling and merging partial data sets from six crystals using XDS^48^ and Aimless^49^ of the CCP4 Suite^50^. For NTSR1-ELF-T4L, a 90.2% complete data set at 2.9 Å resolution was obtained from partial data sets from five crystals.

Structure determination was performed by molecular replacement using the Phaser module of the CCP4 Suite. Two search models were created using the structure of NTSR1-GW5-T4L^3^ (PDB code 4GRV) with one containing the T4 lysozyme domain and one containing the receptor seven-helix bundle. One copy of each search model was found, producing a single solution. Subsequent refinement was performed using the MR solution with rounds of PHENIX.AutoBuild^51^ followed by PHENIX.Refine with simulated annealing, and Refmac5 using translation, libration and screw-rotation (TLS) parameters^52^. TLS displacement groups used in the refinement were defined by the TLSMD server^53^. Manual examination and rebuilding of refined coordinates was accomplished using COOT^54^. The structures were refined with final *R*/*R*_free_ values of 0.23/0.28 for both NTSR1-LF-T4L and NTSR1-ELF-T4L. The quality of the model was checked using the Molprobity server^55^. The Ramachandran statistics for NTSR1-LF-T4L are: 95.3% favoured regions, 4.7% allowed regions, 0% outliers. The Ramachandran statistics for NTSR1-ELF-T4L are: 94.4% favoured regions, 5.6% allowed regions, 0% outliers. The signal-to-noise ratio of *I*/*σ*(*I*)≥2.0 was at 2.8 and 3.1 Å for NTSR1-LF-T4L and NTSR1-ELF-T4L, respectively. A summary of data collection and refinement statistics is reported in Table 1. Simulated annealing omit maps for H8 were generated using the Composite Omit Map function of the Phenix program suite (Supplementary Fig. 6). A stereo figure of representative electron density for TM7 of NTSR1-LF-T4L and NTSR1-ELF-T4L is given in Supplementary Fig. 7.

For NTSR1-ELF-T4L, initial crystal screening was done at the Stanford Synchrotron Radiation Lightsource, beamline 12-2.

Figures were prepared in PyMOL (Schrödinger). Structural alignments were done with the ‘align’ command of PyMOL. Sigma-A weighted maps imported into PyMOL were generated by using the SigmaA program of the CCP4 suite and then converted to maps using the Phenix.mtz2map command line tool.

## Additional information

**How to cite this article:** Krumm, B. E. *et al.* Structural prerequisites for G-protein activation by the neurotensin receptor. *Nat. Commun.* 6:7895 doi: 10.1038/ncomms8895 (2015).

## Supplementary Material

Supplementary Information Supplementary Figures 1-7, Supplementary Tables 1-9 and Supplementary References

## Figures and Tables

**Table 1:** Data collection and refinement statistics.

	**NTSR1-LF-T4L**	**NTSR1-ELF-T4L**
*Data collection*		
Space group	P 2_1_22_1_	P 2_1_22_1_
Mol/ASU	1	1
Cell dimensions		
*a, b, c* (Å)	49.8, 88.4, 161.3	49.1, 88.1, 161.3
α, β, γ (°)	90, 90, 90	90, 90, 90
Resolution (Å)	34.0–2.60 (2.69–2.60)*	45.0–2.90 (3.00–2.90)*
*R*_merge_ (%)	0.15 (0.73)	0.15 (0.80)
Mean *I*/*σ*(*I*)	10.9 (1.5)	9.3 (1.5)
Completeness (%)	99.4 (98.6)	90.2 (87.4)
Redundancy	7.2 (4.0)	6.6 (3.7)
		
*Refinement*		
Resolution (Å)	34.0–2.60	45.0–2.90
No. of total reflections	161,974 (8,709)	96,173 (5,081)
No. of unique reflections	22,562 (2,166)	14,602 (1,371)
*R*_work_/*R*_free_ (%)	23.2/28.0	23.1/28.1
No. of atoms		
Protein	3,714	3,666
Ligand	58	58
Water	55	10
*B*-factors (Å^2^)		
NTSR1-T4L	59.8	71.8
NTSR1	66.5	78.9
T4L	47.7	58.7
NTS_8–13_	56.6	65.5
Water	53.4	54.1
R.m.s. deviations		
Bond lengths (Å)	0.007	0.006
Bond angles (°)	1.185	1.01

R.m.s., root mean squared.

Number of crystals for NTSR1-LF-T4L and NTSR1-ELF-T4L was 6 and 5, respectively.

^*^Highest resolution shell is shown in parenthesis.
