# Supplementary material for: Structural prerequisites for G-protein activation by the neurotensin receptor
Source: Nat Commun. 2015 Jul 24;6:7895. doi: 10.1038/ncomms8895 (PMC4515772; doi:10.1038/ncomms8895)
Supplement: Supplementary Information — Supplementary Figures 1-7, Supplementary Tables 1-9 and Supplementary References [file ncomms8895-s1.pdf]

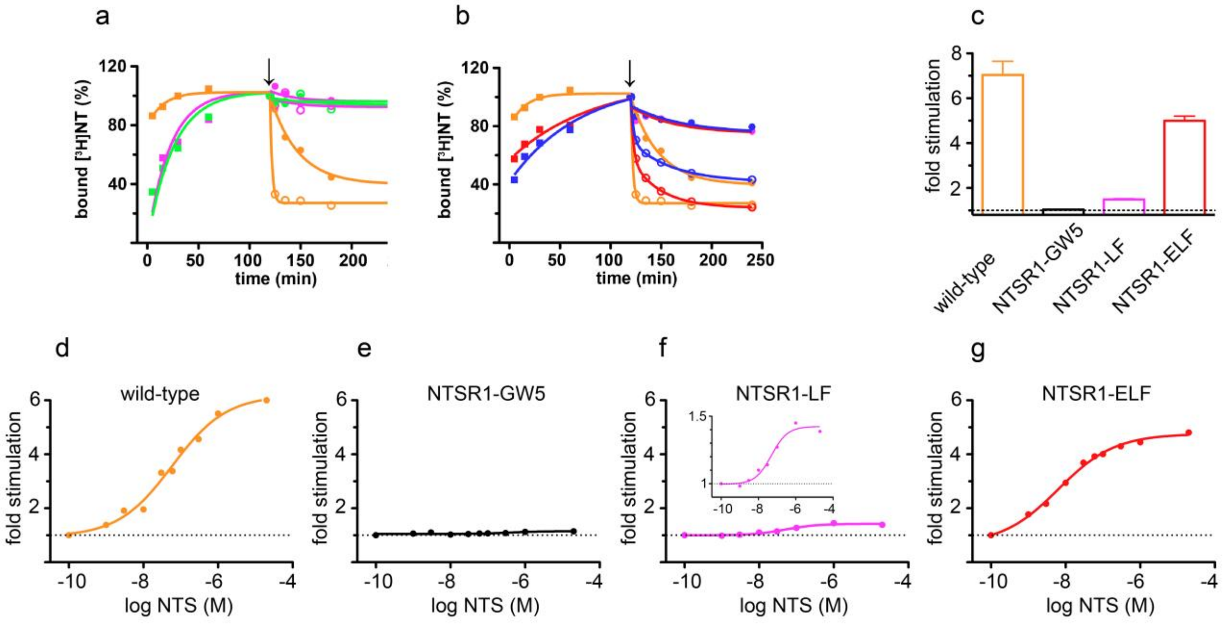

Supplementary Figure 1 | Pharmacological properties of NTSR1 mutants. All binding experiments were conducted with NTSR1 constructs in urea-washed P2 insect cell membranes. All panels have an identical color code: Wild-type NTSR1 (orange), NTSR1-GW5 (black), NTSR1-LF-T4L (green), NTSR1-LF (purple), NTSR1-ELF-T4L (blue), NTSR1-ELF (red). In NTSR1-LF-T4L and NTSR1-ELF-T4L, most of ICL3 is replaced with T4L, but NTSR1-GW5 (ref. 1), NTSR1-EL, and NTSR1-ELF contain an ICL3 identical to that in wild-type NTSR1. (**a-b**) Agonist association and dissociation: [^{3}H]NTS association (closed squares) was probed in TEBB buffer. Dissociation of agonist from NTSR1 was determined by quantifying the amount of [^{3}H]NTS remaining bound to receptors upon addition (arrow) of excess unlabeled NTS in the presence (open circles) or absence of NaCl (closed circles). Representative experiments conducted in singles are shown. Two independent experiments for each construct gave similar results. Wild-type NTSR1 data are taken from ref. 1. (**c**) Agonist-stimulated activation of Gq: GDP/[^{35}S]GTPγS exchange assays contained purified Gq protein, [^{35}S]GTPγS, insect cell membranes with NTSR1, and saturating concentrations of NTS (20 μM). Fold stimulation of the exchange of GDP for [^{35}S]GTPγS on Gq in the presence of NTS is compared to the nucleotide exchange in the absence of ligand (number of independent experiments: wild-type NTSR1 n=7; NTSR1-GW5 n=1; NTSR1-LF n=7; NTSR1-ELF n=6). A value of 1 (dotted line) indicates the absence of receptor-catalyzed nucleotide exchange. Error bars correspond to s.e.m. (**d-g**)

Agonist-stimulated activation of Gq in response to the indicated amounts of NTS. Representative experiments conducted in singles are shown. The results of repeat experiments are shown in Supplementary Table 2.

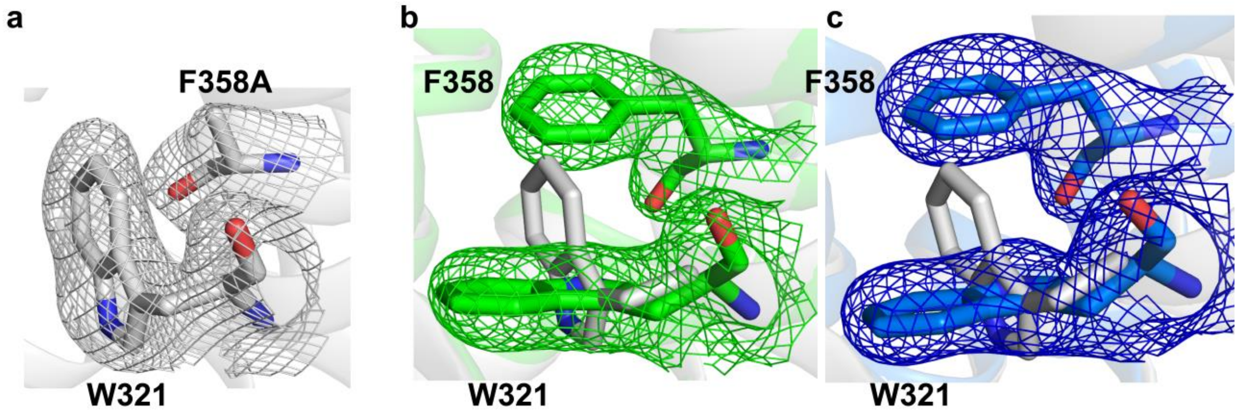

Supplementary Figure 2 | Residue W321$^{6.48}$ of the CWxP motif is oriented parallel to the bilayer plane. (**a**) NTSR1-GW5-T4L (PDB code 4GRV) is colored in grey with mesh outlining the electron density of W321$^{6.48}$ and the thermostabilizing F358A$^{7.42}$ residue. W321$^{6.48}$ is positioned approximately perpendicular to the lipid bilayer. (**b**) NTSR1-LF-T4L is colored in green; the W321$^{6.48}$ is oriented parallel to the bilayer plane as the F358$^{7.42}$ side chain prevents W321$^{6.48}$ from adopting a rotamer conformation as that seen in NTSR1-GW5-T4L. (**c**) NTSR1-ELF-T4L is colored in blue. The Sigma-A weighted 2mFo-DFc maps are contoured at 1σ. The amino acid residues are labeled and depicted as sticks.

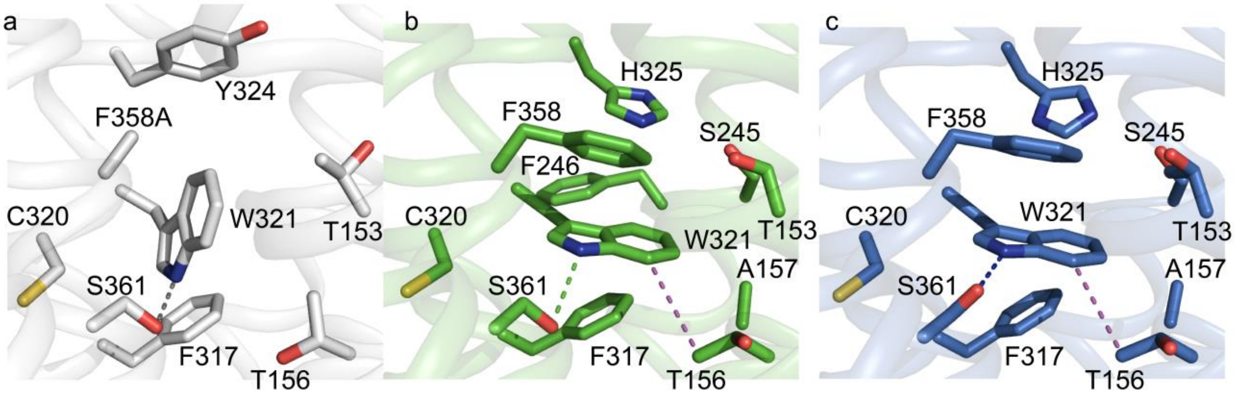

Supplementary Figure 3 | Interaction of W321$^{6.48}$ with NTSR1 residues. (**a**) NTSR1-GW5-T4L (PDB code 4GRV), (**b**) NTSR1-LF-T4L, and (**c**) NTSR1-ELF-T4L are shown in grey, green, and blue, respectively. Individual residues are shown as stick model and are labeled. In contrast to NTSR1-GW5-T4L, the W321$^{6.48}$ side chains of NTSR1-LF-T4L and NTSR1-ELF-T4L form van der Waals interactions with A157$^{3.40}$ and F317$^{6.44}$, residues that have been implicated in helix packing changes upon agonist binding of β_2AR[2]. A purple dashed line indicates an additional hydrophobic contact with T156$^{3.39}$. A stabilizing hydrogen bond between the W321$^{6.48}$ indole ring nitrogen and the S361$^{7.45}$ side chain is seen in all active-like NTSR1 structures.

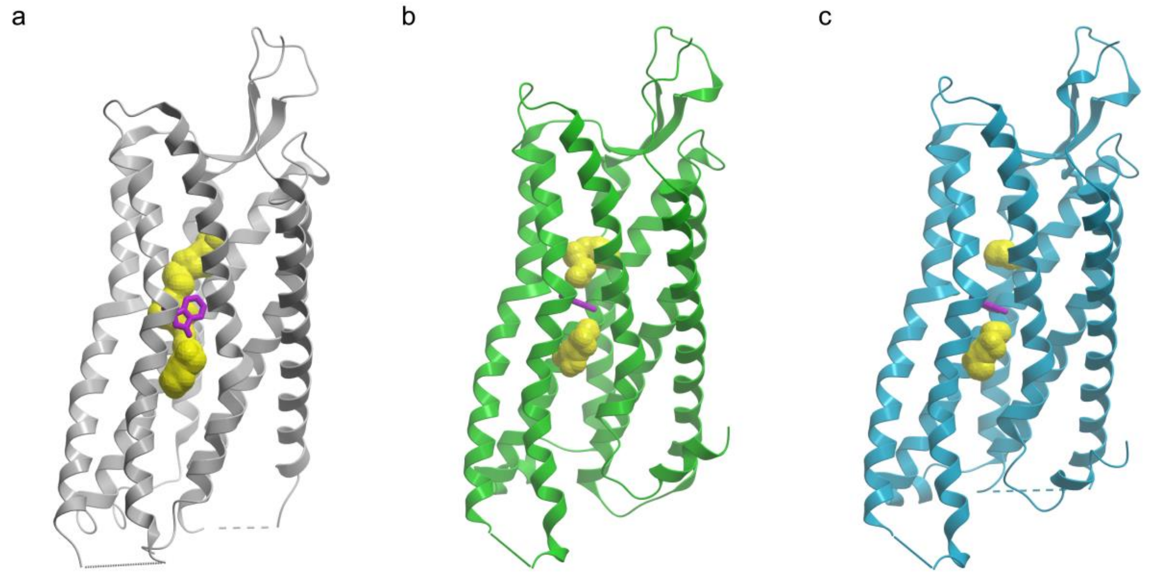

Supplementary Figure 4 | The collapsed Na^+ ion binding pocket. (**a**) NTSR1-GW5-T4L (grey, PDB code 4GRV), (**b**) NTSR1-LF-T4L (green), (**c**) NTSR1-ELF-T4L (blue). In NTSR1-LF-T4L and NTSR1-ELF-T4L, W321$^{6.48}$ has closed off the collapsed Na^+ ion binding pocket by disrupting an extended cavity seen in NTSR1-GW5-T4L. The cavities were displayed using the Closed Cavities function of the ICM-Pro software (Molsoft) and are colored in yellow. W321$^{6.48}$ is shown as stick model in purple.

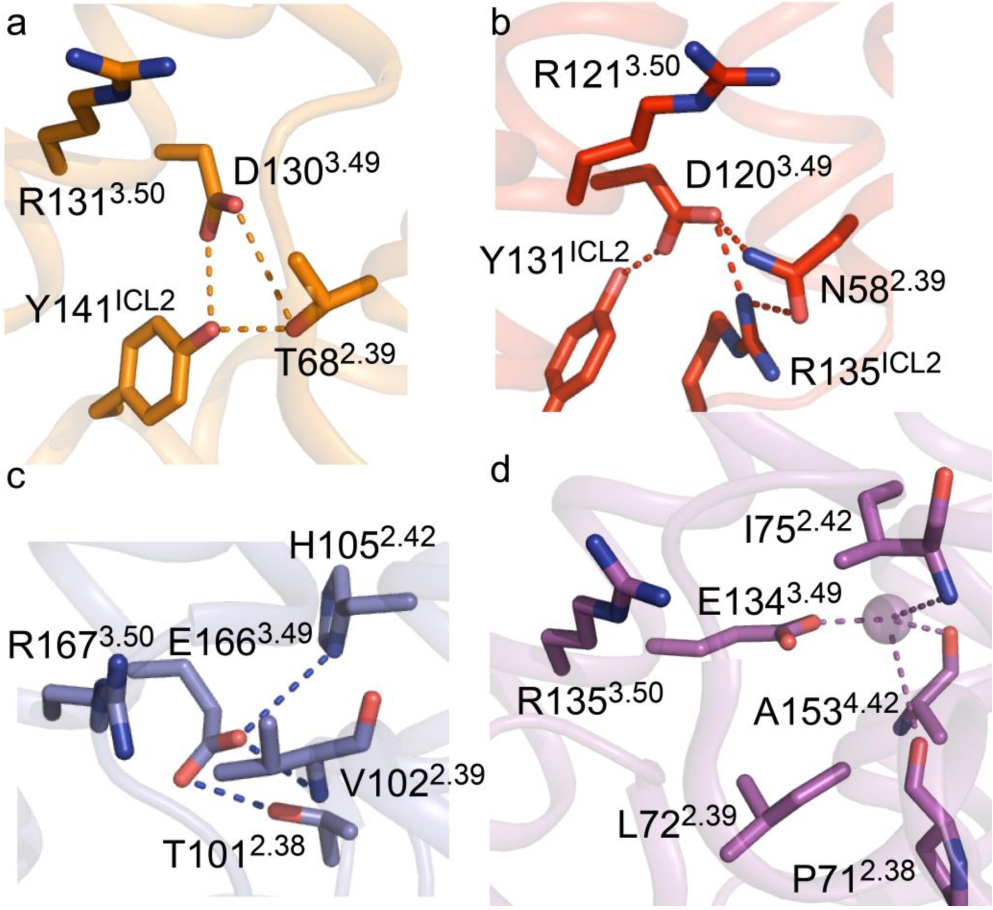

Supplementary Figure 5 | E166$^{3.49}$ contacts. (**a**) β_2AR-Gs complex[3] (orange, PDB code 3SN6); (**b**) active M2 receptor[4] (red, PDB code 4MQS); (**c**) NTSR1-ELF-T4L (blue); (**d**) metarhodopsin II (ref. 5) (purple, PDB code 3PQR). Residues are labeled as stick models, and hydrogen bonds / polar contacts are indicated by dotted lines. E166$^{3.49}$ of NTSR1-ELF-T4L is stabilized by hydrogen bonds to T101$^{2.38}$, V102$^{2.39}$, and H105$^{2.42}$ in TM2. In β_2AR and M2 receptor, D$^{3.49}$ contacts T68$^{2.39}$ and N58$^{2.39}$, respectively. E164$^{3.49}$ of metarhodopsin II is stabilized by water mediated contacts to TM2 and TM4. Receptor residues T68$^{2.39}$ and D130$^{3.49}$ of β_2AR interact with ICL2 via Y141, and residues N58$^{2.39}$ and D120$^{3.49}$ of M2 receptor interact with ICL2 via Y131 and R135.

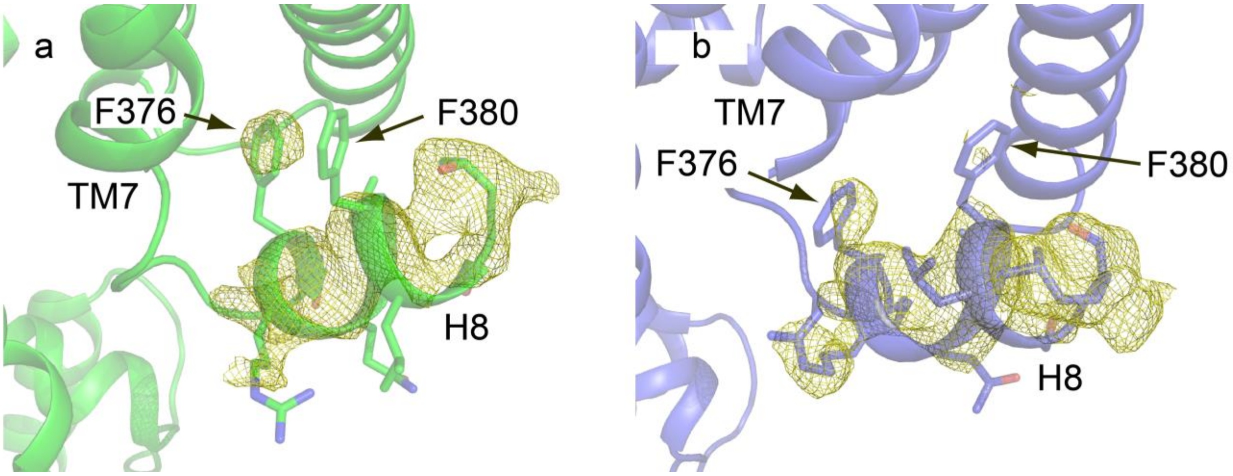

Supplementary Figure 6 | Simulated annealing omit maps for H8. (**a**) NTSR1-LF-T4L (**b**) NTSR1-ELF-T4L. Simulated annealing mFo-DFc difference density omit maps contoured at 3σ were generated using the Phenix Composite Omit Map function by omitting residues A374-T383 or A374-L384 for NTSR1-LF-T4L or NTSR1-ELF-T4L, respectively. Cartesian dynamics at 5000K along with harmonic restraints were applied to the omitted region. Figures were created using PyMOL.

a

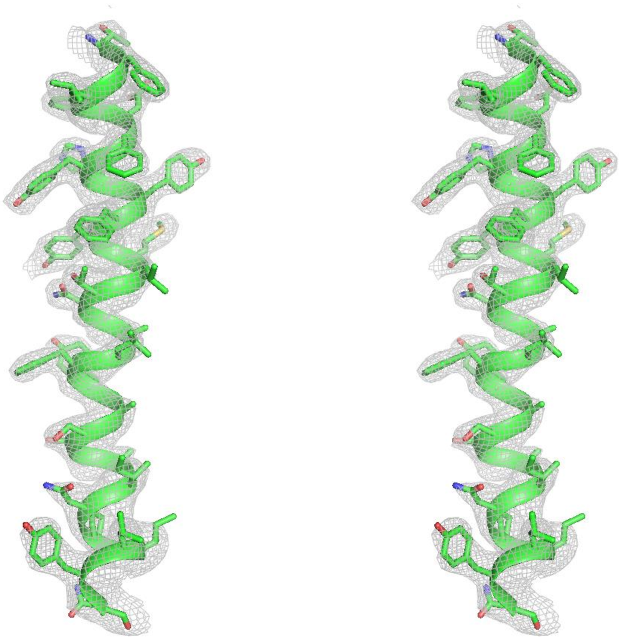

b

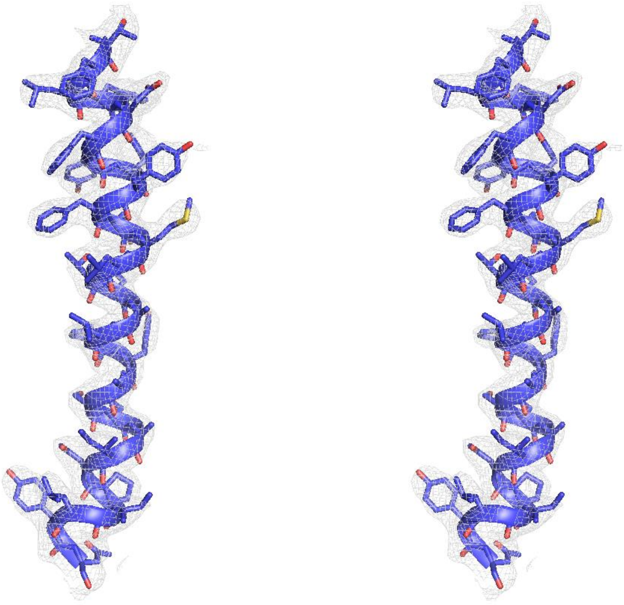

Supplementary Figure 7 | Representative electron density of TM7. (a) NTSR1-LF-T4L (green); **(b)** NTSR1-ELF-T4L (blue). Stereoview of SigmaA weighted 2mFo-DFc maps contoured at 1σ. Maps were generated using the SigmaA program of the CCP4 suite and the Phenix mtz2maps function. Figures were created by using maps imported into PyMOL.

Supplementary Table 1 | NTSR1 constructs used for pharmacological analyses. NTSR1-GW5 contained the six thermostabilizing mutations A86L$^{1.54}$, E166A$^{3.49}$, G215A^{ECL2}, L310A$^{6.37}$, F358A$^{7.42}$, and V360A$^{7.44}$ (ref. 1). In this study, we reverted the mutations E166A$^{3.49}$, L310A$^{6.37}$, and F358A$^{7.42}$ to their wild-type amino acid residues, either alone or in combinations. The letters in the names of the NTSR1 constructs indicate the wild-type residue present i.e. NTSR1-E has a glutamic acid at position 166 (E166$^{3.49}$), but contains the remaining five mutations A86L$^{1.54}$, G215A^{ECL2}, L310A$^{6.37}$, F358A$^{7.42}$, and V360A$^{7.44}$. All constructs consisted of the hemagglutinin signal peptide and the Flag tag, followed by NTSR1 (T43-K396, containing the respective mutations) with the wild-type ICL3 sequence. A deca-histidine tag was placed at the C-terminus. [*], Ballesteros-Weinstein numbers[6]. The sense primers used for QuikChange mutagenesis (Agilent) are:

A166E: 5’-GTAGCCAGCCTGAGTGTGgagCGCTACTTGGCCATCTGCC

A310L: 5’-CGCCACGGAGTCCTCGTCttaCGTGCTGTGGTCATTGCC

A358F: 5’-CTAACCAACGCTCTCttcTACgccAGCTCCGCCATCAATCCC

NTSR1 constructs						
Position	1.54[*]	3.49[*]	ECL2[*]	6.37[*]	7.42[*]	7.44[*]
-GW5	A86L	E166A	G215A	L310A	F358A	V360A
-E	A86L	E166	G215A	L310A	F358A	V360A
-L	A86L	E166A	G215A	L310	F358A	V360A
-F	A86L	E166A	G215A	L310A	F358	V360A
-EL	A86L	E166	G215A	L310	F358A	V360A
-EF	A86L	E166	G215A	L310A	F358	V360A
-LF	A86L	E166A	G215A	L310	F358	V360A
-ELF	A86L	E166	G215A	L310	F358	V360A

Supplementary Table 2 | Pharmacological properties of NTSR1-LF and NTSR1-ELF constructs. All binding experiments were conducted with NTSR1 constructs in urea-washed P2 insect cell membranes. NTSR1-LF-T4L with T4L replacing most of ICL3 was compared to NTSR1-LF containing ICL3; NTSR1-ELF-T4L with T4L replacing most of ICL3 was compared to NTSR1-ELF containing ICL3. NTSR1-GW5-T4L, NTSR1-GW5 (ref. 1), and wild-type NTSR1 were included as reference. All values are given ± s.e.m. from independent experiments conducted as single data points. All data were best fit to equations with a Hill slope of 1, except when indicated otherwise. K_d, equilibrium dissociation constant (saturation binding) but see note below; K_i, dissociation constant calculated by the Cheng and Prusoff equation; $IC_{50\ (NTS)}$, half maximal inhibitory concentration of NTS on [^{3}H]NTS binding (homologous competition); $IC_{50\ (SR48692)}$, half maximal inhibitory concentration of SR48692 on [^{3}H]NTS binding (heterologous competition); $IC_{50\ (NaCl)}$, half maximal inhibitory concentration of Na^+ ions on [^{3}H]NTS binding; EC_{50}, half maximal effective concentration of NTS on the exchange of GDP for GTPγS on Gq; fold stimulation by NTS, fold stimulation of the exchange of GDP for GTPγS on Gq in the presence of saturating concentrations of NTS compared to nucleotide exchange in the absence of ligand. Ligand depletion was below 25% for saturation binding experiments, and below 10% for competition and NaCl titration experiments. For GDP/GTPγS exchange experiments, initial rates of reaction were approximated throughout, that is, <10% of [^{35}S]GTPγS was consumed. Note that all binding experiments using NTSR1-LF-T4L, NTSR1-LF, NTSR1-ELF-T4L, NTSR1-ELF, as well as NTSR1-GW5-T4L and NTSR1-GW5 did not reach equilibrium within the incubation time because of the slow [^{3}H]NTS off-rates. n, number of independent experiments; n.a., not analyzed; n.d., value not determined; [a] value for apparent dissociation constant; [b] data from ref. 1; [c] four parameter variable slope equation (Hill slope = 0.7 ± 0.1 s.e.m, n=4 for wild-type NTSR1 and Hill slope = 0.6 ± 0.1 s.e.m, n=3 for NTSR1-ELF).

	**Wild-type**	**GW5-T4L**	**GW5**
K_d (nM)	1.9 ± 0.3 (n=3)	2.9 ± 0.1 (n=3)[a,b]	2.5 ± 0.2 (n=3)[a,b]
K_i (nM)	1.8 ± 0.5 (n=3)	n.a.	n.a.
$IC_{50\ (NTS)}$ (nM)	6.3 ± 1.4 (n=3)	n.a.	n.a.
$IC_{50\ (SR48692)}$ (nM)	19 ± 7 (n=3)	1800 ± 300 (n=3)[b]	1200 ± 300 (n=3)[b]
$IC_{50\ (NaCl)}$ (mM)	56 ± 3 (n=3)	210 ± 50 (n=3)[b]	180 ± 40 (n=3)[b]

EC_{50} (nM)	37 ± 13 (n=4)[c]	n.d. (n=1)	n.d. (n=1)	
fold stimulation by NTS	7.0 ± 0.6 (n=7)	n.d. (n=3)	n.d. (n=1)	

	**LF-T4L**	**LF**	**ELF-T4L**	**ELF**
K_d (nM)	4.0 ± 0.9 (n=3)[a]	4.3 ± 0.8 (n=5)[a]	1.7 ± 0.1 (n=3)[a]	1.3 ± 0.1 (n=4)[a]
K_i (nM)	n.a.	2.4 ± 0.7 (n=3)	2.1 ± 0.1 (n=3)	1.1 ± 0.3 (n=3)
$IC_{50\ (NTS)}$ (nM)	n.a.	4.0 ± 0.3 (n=3)	11 ± 3 (n=3)	6.4 ± 0.8 (n=3)
$IC_{50\ (SR48692)}$ (nM)	88 ± 8 (n=3)	79 ± 6 (n=3)	66 ± 5 (n=3)	49 ± 10 (n=3)
$IC_{50\ (NaCl)}$ (mM)	1600 ± 200 (n=3)	600 ± 230 (n=3)	360 ± 40 (n=3)	430 ± 80 (n=3)
EC_{50} (nM)	n.d. (n=1)	37 ± 10 (n=3)	n.d. (n=1)	4.7 ± 0.8 (n=3)[c]
fold stimulation by NTS	n.d. (n=2)	1.5 ± 0.1 (n=7)	n.d. (n=1)	5.0 ± 0.2 (n=6)

Supplementary Table 3 | Denaturation temperatures (T_m) of NTSR1 mutants and wild-type NTSR1 in detergent solution. Denaturation profiles were recorded in the presence of [^{3}H]NTS and the detergent mixture LMNG-CHS. All values are given ± s.e.m. from 3 independent experiments conducted as single data points. Data for wild-type NTSR1 (Met-T43-Y424) and NTSR1-GW5-T4L are from ref. 1. [a] The means of the apparent T_m values are significantly different (unpaired, two-tailed t test; P<0.0001).

	**Apparent T_m (ºC)**	**ΔT_m (ºC)**
Wild-type NTSR1	40 ± 1	
NTSR1-GW5-T4L[a]	59 ± 0	19
NTSR1-GW5[a]	65 ± 0	25
NTSR1-LF-T4L	53 ± 1	13
NTSR1-LF	56 ± 1	16
NTSR1-ELF-T4L	51 ± 1	11
NTSR1-ELF	54 ± 1	14

Supplementary Table 4 | Hydrogen bonds and salt bridges between NTS_{8-13} and NTSR1-LF-T4L. The analysis was performed using the PDBePISA server[7]. Abbreviations and symbols used are as follows: H, hydrogen bond; S, salt bridge; TM, transmembrane helix; N, amino-terminus; ECL, extracellular loop.

**NTS_{8-13} atoms**		**Distance (Å)**	**NTSR1 atoms**		**Location in NTSR1**
Arg8	[NH1]	2.7 (H)	Asp56	[O]	N
Arg8	[NH1]	3.4 (H)	Asp54	[O]	N
Arg8	[NE]	3.1 (H)	Asp54	[O]	N
Arg8	[NH2]	3.7 (H)	Thr341	[OG1]	TM7
Arg8	[NH1]	3.2 (H)	wat2		
Arg8	[NH2]	2.7 (H)	wat2		
Asp345		2.9 (H)	wat2	[OD1]	TM7
Asp345		3.7 (H)	wat2	[OD2]	TM7
Arg9	[NH1]	2.9 (H)	Phe331	[O]	TM6
Arg9	[NH1]	2.6 (H)	Ile334	[O]	ECL3
Arg9	[NH1]	3.9 (H)	Cys332	[O]	TM6
Arg9	[NH2]	3.1 (H,S)	Asp336	[OD1]	ECL3
Arg9	[NE]	2.8 (H)	wat7		
Pro10	[O]	2.6 (H)	wat7		
Tyr11	[OH]	2.8 (H)	Leu55	[O]	N
Tyr11	[OH]	3.4 (H)	His132	[O]	ECL1
Tyr11	[OH]	3.9 (H)	His133	[N]	ECL1
Ile12	[O]	2.9 (H)	Tyr347	[OH]	TM7
Leu13	[O]	2.8 (H,S)	Arg327	[NH1]	TM6
Leu13	[O]	3.6 (S)	Arg327	[NH2]	TM6
Leu13	[O]	2.9 (H)	wat9		
Leu13	[O]	3.5 (H)	wat10		
Leu13	[OXT]	2.6 (H)	Tyr146	[OH]	TM3
Leu13	[OXT]	3.5 (H)	wat9		
Leu13	[OXT]	3.3 (H)	wat10		
Arg328		2.5 (H)	wat9	[NH1]	TM6
Tyr351		2.5 (H)	wat10	[OH]	TM7

Supplementary Table 5 | Potential intermolecular interactions of NTSR1-LF-T4L residues and NTS$_{8-13}$. The analysis was performed using the PDBePISA server. Abbreviations used are as follows: TM, transmembrane helix; N, amino-terminus; ECL, extracellular loop; hydrogen bonds are indicated in blue.

NTSR1 atoms		Location in NTSR1
Ser53	[O]	N
Asp54	[O] [CA] [CB] [CG] [OD2] [C]	N
Leu55	[O] [CA] [CB] [CD2]	N
Asp56	[O] [CB] [C]	N
Val57	[CG2]	N
Asn58	[N] [CB]	N
Asn127	[O]	TM2
Phe128	[O] [CA] [CB] [CD1] [CE1] [CE2]	TM2
His132	[O] [C] [CA] [CB] [CD2]	ECL1
His133	[N] [CB]	ECL1
Tyr146	[CE1] [CZ] [OH]	TM3
Met204	[SD] [CE]	TM4
Met208	[SD] [CE]	ECL2-β1
Arg213	[CD] [NE] [CZ] [NH2]	ECL2
Val224	[CB] [CG1] [CG2]	ECL2-β2
Cys225	[O]	ECL2-β2
Thr226	[CA] [CB] [OG1]	ECL2-β2
Pro227	[CG] [CD]	ECL2-β2
Thr231	[OG1] [CG2]	ECL2
Val234	[CG1]	TM5
Ile238	[CG1]	TM5
Arg327	[NH1] [NH2]	TM6
Arg328	[CA] [CB] [CG] [CZ] [NH1] [NH2] [O]	TM6
Phe331	[O] [CA] [CB] [CG] [CD1] [CE1] [CZ] [CE2] [CD2]	TM6
Cys332	[CA] [O]	TM6
Ile334	[O]	ECL3
Ser335	[CA]	ECL3
Asp336	[CB] [CG] [OD1] [OD2] [O]	ECL3
Trp339	[O] [CB] [CG] [CD1] [CD2] [NE1] [CE2] [CE3] [CZ3] [CH2] [CZ2] [C]	ECL3
Thr340	[CA] [O] [C]	ECL3
Thr341	[N] [CA] [OG1] [CG2]	TM7
Phe344	[CA] [CB] [CG] [CD1] [CD2] [CE1] [CE2] [CZ]	TM7
Asp345	[OD1]	TM7

Tyr347	[CB] [CG] [CD1] [CD2] [CE1] [CE2] [CZ] [OH]	TM7
His348	[CD2] [CE1] [NE2]	TM7
Tyr351	[CG] [CD1] [CE1] [CD2] [CE2] [CZ] [OH]	TM7

Supplementary Table 6 | Hydrogen bonds and salt bridges between NTS_{8-13} and NTSR1-ELF-T4L. The analysis was performed using the PDBePISA server. Abbreviations and symbols used are as follows: H, hydrogen bond; S, salt bridge; TM, transmembrane helix; N, amino-terminus; ECL, extracellular loop.

**NTS_{8-13} atoms**		**Distance (Å)**	**NTSR1 atoms**		**Location in NTSR1**
Arg8	[NH1]	2.7 (H)	Asp56	[O]	N
Arg8	[NH1]	2.8 (H)	Asp54	[O]	N
Arg8	[NE]	2.8 (H)	Asp54	[O]	N
Arg9	[NH1]	2.4 (H)	Phe331	[O]	TM6
Arg9	[NH2]	3.0 (H)	Ile334	[O]	ECL3
Tyr11	[OH]	3.8 (H)	His133	[N]	ECL1
Tyr11	[OH]	2.8 (H)	Leu55	[O]	N
Tyr11	[O]	2.7 (H)	Thr226	[OG1]	ECL2
Ile12	[O]	2.6 (H)	Tyr347	[OH]	TM7
Leu13	[O]	3.1 (H,S)	Arg327	[NH1]	TM6
Leu13	[O]	3.2 (H,S)	Arg327	[NH2]	TM6
Leu13	[O]	2.8 (H,S)	Arg328	[NH1]	TM6
Leu13	[OXT]	2.7 (H)	Tyr146	[OH]	TM3
Leu13	[OXT]	3.0 (H)	Tyr351	[OH]	TM7

Supplementary Table 7 | Potential intermolecular interactions of NTSR1-ELF-T4L residues and NTS_{8-13}. The analysis was performed using the PDBePISA server. Abbreviations used are as follows: TM, transmembrane helix; N, amino-terminus; ECL, extracellular loop; hydrogen bonds are indicated in blue.

NTSR1 atoms		Location in NTSR1
Ser53	[O]	N
Asp54	[O] [CA] [CB] [CG] [OD2] [C]	N
Leu55	[O] [CA] [CB] [CD2]	N
Asp56	[O] [CB]	N
Val57	[CG2]	N
Asn58	[CB] [N]	N
Phe128	[O] [CA] [CB] [CD1] [CE1] [CE2]	TM2
His132	[O] [C] [CA] [CB] [CD2]	ECL1
His133	[CB]	ECL1
Tyr146	[CE1] [CZ] [OH]	TM3
Arg149	[CD] [CZ]	TM3
Met204	[CE]	TM4
Met208	[SD] [CE]	ECL2-β1
Arg213	[CD] [CZ] [NE] [NH2]	ECL2
Val224	[CB] [CG1] [CG2]	ECL2-β2
Cys225	[O]	ECL2-β2
Thr226	[CA] [CB] [OG1]	ECL2-β2
Pro227	[CG] [CD]	ECL2-β2
Ile238	[CG1] [CD1]	TM5
Arg327	[NH1] [NH2]	TM6
Arg328	[CA] [CG] [CZ] [NH1] [NH2] [O] [NE]	TM6
Phe331	[O] [CB] [CG] [CD1] [CE1] [CZ] [CE2] [CD2]	TM6
Cys332	[CA] [O]	TM6
Ile334	[O]	ECL3
Ser335	[CB] [OG]	ECL3
Asp336	[CG] [OD1] [OD2] [O]	ECL3
Trp339	[CB] [CG] [CD1] [NE1] [CE2] [CD2] [CE3] [CZ3] [CH2] [CZ2] [C] [O]	ECL3
Thr340	[CA] [C] [N]	ECL3
Thr341	[CA] [OG1] [CG2] [N]	TM7
Phe344	[CA] [CB] [CG] [CD1] [CE1] [CZ] [CE2] [CD2]	TM7
Asp345	[OD1]	TM7
Tyr347	[CB] [CG] [CD1] [CE1] [CZ] [OH] [CE2]	TM7
His348	[CD2] [CE1] [NE2]	TM7

Tyr351	[CG] [CD1] [CE1] [CD2] [CE2] [CZ] [OH]	TM7
Asn355	[ND2]	TM7

Supplementary Table 8 | Relative positions of the intracellular ends of TM3, TM5, and TM6 in NTSR1-LF-T4L, NTSR1-ELF-T4L and NTSR1-GW5-T4L. Receptors without the T4 lysozyme were aligned in PyMOL, and the distances measured between the indicated Cα atoms. GW5, NTSR1-GW5-T4L; LF, NTSR1-LF-T4L; ELF, NTSR1-ELF-T4L.

Residue	Distance between a given Cα atom in two structures		Direction compared to NTSR1-GW5
	GW5 – LF	GW5 – ELF	
C172$^{3.55}$ – C172$^{3.55}$	2.7 Å	2.2 Å	outwards
M267$^{5.68}$ – M267$^{5.68}$	3.6 Å	3.3 Å	inwards
L303$^{6.30}$ – L303$^{6.30}$	3.5 Å	3.5 Å	inwards
	Distance between two given Cα atoms in the same structure		
	GW5	LF	ELF
R167$^{3.50}$ – L303$^{6.30}$	14.0 Å	13.6 Å	13.3 Å
M267$^{5.68}$ – L303$^{6.30}$	7.8 Å	9.7 Å	9.5 Å

Supplementary Table 9 | Secondary structure assignment of H8. Hydrogen-bonded turns (T) and α-helices (H) were defined using the Kabsch and Sander algorithm[8] (DSSP server). F376$^{8.50}$ is a conserved residue of the H8 motif. NTSR1-ELF shows electron density (e.d.) including L384; NTSR1-LF shows electron density including T383, and TM86V-ΔIC3A[9] (PDB code 3ZEV) has electron density including C386. * The TM86V-ΔIC3A crystal contains 2 molecules per asymmetric unit.

	A374	N375	F376	R377	Q378	V379	F380	L381	S382	T383	L384	A385	C386
ELF	T	T	H	H	H	H	T	T	T	e.d.	e.d.		
LF	T	T	H	H	H	H	H	H	T	e.d.			
3ZEV	H	H	H	H	H	H	H	H	H	H	H/T*	H/T*	e.d.

Supplementary References

1. White, J. F. *et al.* Structure of the agonist-bound neurotensin receptor. *Nature* **490**, 508-513 (2012).
2. Rasmussen, S. G. *et al.* Structure of a nanobody-stabilized active state of the beta(2) adrenoceptor. *Nature* **469**, 175-180 (2011).
3. Rasmussen, S. G. *et al.* Crystal structure of the beta2 adrenergic receptor-Gs protein complex. *Nature* **477**, 549-555 (2011).
4. Kruse, A. C. *et al.* Activation and allosteric modulation of a muscarinic acetylcholine receptor. *Nature* **504**, 101-106 (2013).
5. Choe, H. W. *et al.* Crystal structure of metarhodopsin II. *Nature* **471**, 651-655 (2011).
6. Ballesteros, J. A. & Weinstein, H. Integrated methods for the construction of three-dimensional models and computationla probing of structure-function relations in G protein-coupled receptors. *Methods Neurosci.* **25**, 366-428 (1995).
7. Krissinel, E. & Henrick, K. Inference of macromolecular assemblies from crystalline state. *J. Mol. Biol.* **372**, 774-797 (2007).
8. Kabsch, W. & Sander, C. Dictionary of protein secondary structure: pattern recognition of hydrogen-bonded and geometrical features. *Biopolymers* **22**, 2577-2637 (1983).
9. Egloff, P. *et al.* Structure of signaling-competent neurotensin receptor 1 obtained by directed evolution in Escherichia coli. *Proc. Natl. Acad. Sci. U.S.A.* **111**, E655-662 (2014).
